# Evaluation of safety, immunogenicity, and efficacy of inactivated reverse-genetics-based H5N8 highly pathogenic avian influenza virus vaccine with various adjuvants via parenteral and mucosal routes in chickens

**DOI:** 10.3389/fimmu.2025.1539492

**Published:** 2025-03-20

**Authors:** Kairat Tabynov, Aidana Kuanyshbek, Leila Yelchibayeva, Kuantay Zharmambet, Zauresh Zhumadilova, Gleb Fomin, Nikolai Petrovsky, Olaitan C. Shekoni, Gourapura J. Renukaradhya, Kaissar Tabynov

**Affiliations:** ^1^ International Center for Vaccinology, Kazakh National Agrarian Research University, Almaty, Kazakhstan; ^2^ Central Reference Laboratory, M. Aikimbayev National Scientific Center for Especially Dangerous Infections, Almaty, Kazakhstan; ^3^ T&TvaX LLC, Almaty, Kazakhstan; ^4^ National Collection of Deposited Strains, Almaty Branch of National Reference Veterinary Center, Almaty, Kazakhstan; ^5^ Vaxine Pty Ltd, Adelaide, SA, Australia; ^6^ Center for Food Animal Health, Department of Animal Sciences, College of Food Agricultural and Environmental Sciences, The Ohio State University (OSU), Wooster, OH, United States

**Keywords:** avian influenza, vaccine, H5N8, reverse genetics; efficacy, adjuvants, nanoparticles

## Abstract

**Background:**

Highly pathogenic H5Nx avian influenza (HPAI) poses a significant threat to poultry health globally, necessitating the development of effective vaccination strategies.

**Methods:**

This study assessed the immunogenicity and efficacy of a reverse-genetics-derived, Differentiating Infected from Vaccinated Animals (DIVA)-compatible inactivated H5N8 vaccine based on the IDCDC-RG71A strain. The vaccine was formulated with different adjuvants, including Montanide ISA 78 VG, ISA 71 R VG, GEL P PR, and mannose-conjugated chitosan nanoparticles, and administered via either the subcutaneous (SC) or intranasal (IN) route. To evaluate safety, the vaccine was tested in specific antibody negative (SAN) chickens, showing no adverse effects. Immunogenicity was assessed by measuring hemagglutination inhibition (HI) antibody titers, antigen-specific IgA and IgY levels, and CD4+ and CD8+ T cell proliferation. Vaccine efficacy was determined through a challenge study using a field isolate of H5N1.

**Results:**

This showed that a single SC dose of vaccine containing ISA 78 VG or ISA 71 R VG provided the best efficacy against infection, with high survival rates, control of abnormally high temperature incidence, reduced virus shedding, and reduced lung and liver lesions. The ISA 78 VG-adjuvanted SC vaccine induced the highest HI titers and CD4+ T cell proliferation, while ISA 71 R VG and GEL P PR elicited the strongest IgY responses. In contrast, IN formulations induced IgA in the lungs and trachea however, even after two doses, failed to generate high HI titers and provided poor, if any, protection against infection. This highlights the superior efficacy of the SC over the IN route of vaccination for reducing H5N1 viral shedding.

**Conclusion:**

These results underscore the importance of both the adjuvants and delivery route to maximize HPAI vaccine efficacy. This presented system could thereby be used to develop potent and DIVA-compatible vaccines to enhance biosecurity and disease management in regions affected by endemic HPAI.

## 1 Introduction

Outbreaks of novel H5N1, H5N6, and H5N8 highly pathogenic avian influenza (HPAI) viruses of the A/Goose/Guangdong/1/96 (Gs/GD) lineage have been extensively spreading across Asia, Europe, Africa, and North America (1–4). These epidemics pose a serious global threat to the poultry industry, ecosystems, and endangered wild bird species and represent a zoonotic risk to human health (5).

Beginning in late 2020, there have been ongoing reports of outbreaks of the HPAI H5N1 virus (clade 2.3.4.4b) worldwide, affecting both wild birds and, on several occasions, domestic poultry. During this period, significant genetic evolution and reassortment have occurred, leading to the emergence of multiple variants (6). Since the fall of 2020, Kazakhstan (KZ) has experienced a series of large outbreaks of HPAI poultry outbreaks among domestic birds that turned out to be the H5N8 subtype of HPAI clade 2.3.4.4b virus (7, 8). The first reported outbreaks occurred in populated areas along the KZ–Russian border. By the end of 2023, outbreaks had been reported in 11 regions of KZ. The Veterinary Service of Kazakhstan made efforts to slow down the spread of the disease through strict quarantines in the affected settlements, imposed export restrictions on poultry and poultry products, and organized prompt vaccination of poultry (9). The recent outbreak of HPAI in KZ (around Lake Karakol near the Caspian Sea coast in Mangistau Oblast) in wild birds was registered between December 28, 2023, and January 9, 2024, wherein 228 mute swans died (2), and a total of 1,132 swans were reported dead until January 25, 2024 (10). During the HPAI outbreak, our research group isolated and characterized a genetically distinct strain of avian influenza virus, A/mute swan/Mangystau/1-S24R-2/2024 (1-S24R-2), subtype H5N1 (clade 2.3.4.4b), from the lung of a deceased mute swan. The highly pathogenic nature of this strain was confirmed based on the presence of the PLREKRRRRKRGLF polybasic cleavage site in the hemagglutinin (HA) gene (2).

Vaccination remains a cornerstone in controlling the spread of HPAI viruses, with the choice of adjuvant and delivery route playing a critical role in determining vaccine efficacy. Adjuvants enhance vaccine protection by optimizing antigen presentation and recognition and prolonging immune memory. Commonly used adjuvants in birds include oil-based emulsions, aqueous formulations, and nanoparticles, each tailored to specific parenteral or mucosal delivery routes. For example, mineral oil-based adjuvants like Montanide ISA 71 R VG and ISA 78 VG elicit strong humoral and cellular responses, while aqueous-based adjuvants such as Montanide GEL P PR are optimized for ease of administration and reduced reactogenicity (11). Recent advances, including mannose-conjugated chitosan nanoparticles (mCS-NPs), provide targeted mucosal delivery and enhance antigen uptake by dendritic cells, potentially improving adaptive immunity (12).

Vaccination is used to control the spread of H5Nx HPAI virus in bird populations in the Republic of Kazakhstan (KZ). According to the national strategy for control of HPAI in KZ beginning 2019, vaccination of parent stock of birds in poultry farms and in households located within a 20-km zone from the hotspot farms as well as poultry in farms located in areas close to migration of wild birds and high-risk areas was carried out (13). Similar vaccination strategies have been adopted in other countries heavily affected by H5Nx HPAI outbreaks. For instance, China has implemented widespread vaccination programs to control HPAI in domestic poultry, which has significantly reduced virus circulation. Egypt, Mexico, and Vietnam have also utilized targeted vaccination campaigns to manage endemic HPAI outbreaks in high-risk regions (14). These strategies emphasize the importance of vaccination as a global tool to mitigate the economic and ecological impact of HPAI in poultry industries and to reduce the zoonotic risk to humans.

Despite the ongoing mass immunization of flocks against HPAI in KZ poultry farms, cases of mortality and reduced productivity of poultry from HPAI vaccine breakthrough infections still continue to occur (personal communication with veterinarians of poultry farms). The effectiveness of poultry vaccines is influenced by many factors, including the match between the vaccine strain and the antigenic variations in the circulating virus strains (15). According to our analysis (2), the genetic similarity in the nucleotide sequence of the hemagglutinin (HA) gene of vaccine strains widely used in commercial HPAI vaccines in Eurasian Economic Union (EEU) countries and the circulating KZ strain 1-S24R-2 of HPAI virus (clade 2.3.4.4b) is only 90%–92%. However, HPAI vaccine efficacy is not only associated with HA similarity between the vaccine and the virus strain but also depends on any adjuvant used, antigen dose, and immunization route (16).

Previous studies have demonstrated that reverse-genetics-based H5N8 vaccines offer a promising approach for combating HPAI viruses due to their ability to incorporate precise genetic modifications for improved safety and immunogenicity. A study by Gao et al. (2022) highlighted the robust immune responses elicited by an inactivated reverse-genetics-based H5N8 vaccine derived from the A/Astrakhan/3212/2020 strain. This vaccine, formulated with squalene-based adjuvant, induced strong humoral immunity and cross-reactivity across multiple H5 clades in animal models, emphasizing the critical role of adjuvant selection (17). Similarly, the rgH5N2 vaccine provided broad protection in avian H5Nx models. Studies, including those by Panickan et al. (2022), have shown that rgH5N2, either alone or combined with HA stalk antigens, elicits strong hemagglutination inhibiting (HI) activity and neutralizing antibody responses against H5N1, H5N8, and H9N2, reduces viral shedding, and provides protection against lethal H5N1 and H5N8 challenges. However, HA stalk antigens alone proved inadequate against H5N1 and H5N8, highlighting the critical need for strategic antigen selection and optimized vaccine design for broader protection (18). Despite these advances, challenges remain in optimizing the efficacy of these vaccines against evolving clade 2.3.4.4b viruses and ensuring compatibility with diverse immunization strategies. For this study, we assessed a panel of veterinary adjuvants supplied by Seppic. This included Montanide ISA 71 R VG, a water-in-mineral-oil adjuvant based on a mannide-oleate-based surfactant system that can be used at flexible ratios in the vaccine, Montanide ISA 78 VG, a water-in-oil emulsion specifically designed for use in chickens, and Montanide GEL P PR, based on a dispersion of highly stable gel particles of sodium polyacrylate in water that induces a depot effect with slow release due to polymer adsorption properties. In addition, we tested an in-house mannose-conjugated chitosan nanoparticle (mCS-NP) adjuvant as previously described (12).

Our aim in this study was to develop and evaluate different HPAI vaccine formulations using a WHO candidate vaccine virus, IDCDC-RG71A (H5N8; clade 2.3.4.4b; reverse genetics derived reassortant) (19), with a range of injectable and mucosal adjuvants. The safety, immunogenicity, and efficacy of candidate HPAI vaccines (clade 2.3.4.4b) administered via parenteral and mucosal routes were compared to a commercial vaccine available in the EEU countries. The intranasal (IN) route of inoculation was chosen to evaluate its potential to induce localized immunity in the upper respiratory tract, particularly in the nasal and airway mucosa, which are primary sites of avian influenza virus entry and replication. The vaccines were assessed for their ability to protect specific antibody negative (SAN) chickens against H5N1 infection. The study results will facilitate the development of improved vaccines effective against current HPAI threats in KZ, thereby assisting to mitigate the impact of HPAI outbreaks on poultry industries, wildlife, and public health.

## 2 Materials and methods

### 2.1 Facility and biosafety statement

All experiments involving the infectious HPAI virus were conducted in biosafety level 3 (BSL-3) and animal biosafety level 3 (ABSL-3) facilities within the Central Reference Laboratory (CRL) at the M. Aikimbayev National Scientific Center for Especially Dangerous Infections (NSCEDI) under the Ministry of Health of the Republic of Kazakhstan (MoH RK). These facilities are accredited according to ISO 35001:2019, which outlines biorisk management for laboratories and other related organizations. The facility’s security is maintained through procedures approved by the NSCEDI institutional biosafety officers. All aspects of the facilities, including procedures, training records, safety drills, and inventory logs, undergo regular inspections and continuous oversight by the institutional biosafety officers, who work closely with the facility managers. Experienced personnel worked indoors in pairs (following the two-person rule). Staff wore powered air-purifying respirators (PAPRs) that filtered the air when they worked with HPAI in the lab and birds. Researchers were decontaminated before leaving the facility and then showered upon exiting the facility. The research program, including procedures, occupational health plans, security, and facilities, is subject to an annual review by an official from the MoH RK (20). At the conclusion of the experiments, all waste and infected animal carcasses were autoclaved and incinerated to ensure the elimination of biohazards.

### 2.2 Ethics statement

All animal experiments were conducted in full compliance with the ARRIVE guidelines, adhering strictly to the UK Animals (Scientific Procedures) Act of 1986 and associated guidelines as well as the EU Directive 2010/63/EU. The sex of the animals was indicated, and any influence or association of sex on the study’s results was appropriately analyzed and reported. The protocol was approved by the Institutional Animal Care and Use Committee (IACUC) of the NSCEDI (Protocol No. 17, dated November 1, 2022). The birds were kept in specialized cages (three pullets/m²) in a facility with 35%–45% humidity at 22°C –23°C, with air exchange occurring at least 16 times per hour. They were housed on deep bedding with drinkers, which were monitored and changed frequently, and feed was provided *ad libitum*. All birds were kept separately in groups (i.e., one group per room) in the ABSL-3 laboratory of the CRL. The birds received daily veterinary supervision, conditions were maintained to ensure a normal state of health, opportunities were provided to meet their physiological and behavioral needs, and factors that could cause stress and distress were rapidly eliminated. Following the challenge with HPAI infection, all surviving pullets were euthanized by administering sodium pentobarbital (5 g/mL). Humane endpoint criteria for birds after infection comprised of greater than or equal to 35% body weight loss or inability to remain upright.

### 2.3 Viruses, cells, and birds

The influenza virus strain A/mute swan/Mangystau/1-S24R-2/2024 (H5N1; clade 2.3.4.4b; 1-S24R-2; GISAID accession number: EPI_ISL_18898050; GenBank accession numbers: PP267962, PP267963, PP267964, PP267965, PP267966, PP267967, PP267968, and PP267969) was isolated in 10-day-old SAN embryonated chicken eggs (ECEs) from the lung of a dead mute swan found in Lake Karakol (Kazakhstan) during a HPAI outbreak in 2024 (2).

The influenza virus IDCDC-RG71A (H5N8; clade 2.3.4.4b; RG71A; Lot A2021JUL06) is a reverse-genetics-derived reassortant (19). The RG71A virus is composed of six gene segments (PB2, PB1, PA, NP, M, and NS) that encode A/Puerto Rico/8/1934 (H1N1) proteins. The HA gene segment and neuraminidase (NA) gene segment of RG71A were derived from A/Astrakhan/3212/2020 (H5N8; GenBank HA: OM403993; GenBank NA: OM403994) with HA protein modified to contain a protease cleavage site characteristic of low pathogenic AI viruses. RG71A influenza virus was generated under a quality system using qualified Vero cells per WHO guidance and excluded from the select agent list by the United States Department of Agriculture on August 12, 2021 to enable use under USDA APHIS BSL-2 permit for candidate vaccine virus. The virus RG71A was kindly provided by the Centers for Disease Control and Prevention (CDC, USA) as part of the WHO’s Pandemic Influenza Preparedness Framework. The 50% egg infective dose (EID_50_) of the virus was measured, and aliquots of allantoic fluid were stored at −80°C until use.

Madin-Darby Canine Kidney (MDCK) cells (ATCC^®^ CCL-34™, NBL-2) were grown in Dulbecco’s modified Eagle’s medium (DMEM; Gibco, UK) supplemented with heat-inactivated 10% fetal bovine serum (FBS; Gibco, UK) and antibiotics (100 units/mL penicillin and 100 μg/mL streptomycin; Gibco, UK) at 37°C in a 5%-CO_2_ incubator. MDCK cells in 96-well tissue culture plates were used to measure viral titers in tracheal and cloacal swab samples after challenge infection using the Reed and Mench method (21) expressed in log_10_ TCID_50_/0.2 mL.

Four-week-old SAN White Leghorn pullets (*G. gallus domesticus*) purchased from a commercial poultry farm in Almaty, Kazakhstan, were used in this experiment. The pullets had not been vaccinated in the poultry farm and were subjected to serological testing upon arrival at the ABSL-3 facility using the hemagglutination inhibition (HI) assay with 1% chicken red blood cells (RBCs). All pullets tested seronegative for H5 AIV.

### 2.4 Preparation of experimental vaccine formulations

The candidate vaccine strain RG71A was inoculated containing 10^4^ EID_50_ of virus in 0.1 mL into the allantoic cavity of 10-day-old SAN embryonated chicken eggs (ECEs) and incubated at 34°C. The allantoic fluids were harvested at 48 h post-infection and clarified by centrifugation at 1,800 × *g* for 30 min at 4°C, and the titer was determined using EID_50_ and hemagglutination (HA) assays. The clarified allantoiс fluid of RG71A virus was inactivated with 0.1% formaldehyde (Sigma, Germany) for 30 h at 37°C, and neutralization of formaldehyde was carried out with sodium bisulfite (NaHSO_3_) at a final concentration of 0.4%. Complete inactivation of the candidate vaccine virus was confirmed by inoculating the virus into the allantoic fluid of 10-day-old ECEs, followed by the hemagglutination (HA) assay using 1% chicken red blood cells (RBCs). We used HA units to measure the functional activity of the hemagglutinin (HA) protein rather than the HA mass in micrograms (µg). This approach reflects the functional capacity of the HA antigen and is used for standardization of influenza vaccine doses. The inactivated candidate vaccine strain “antigen” was mixed with Montanide ISA 71 R VG, Montanide ISA 78 VG, and Montanide GEL P PR (Seppic, France) for parenteral administration, and for mucosal IN delivery, mannose-conjugated chitosan nanoparticles (mCS-NPs) and Montanide GEL P PR adjuvants were used according to the vaccine preparation protocols from the manufacturer (**Table 1**). Commercial vaccine VOLVAC^®^B.E.S.T AI+ND (**Table 1**; Group 4) produced by Boehringer Ingelheim Vetmedica, S.A. DEC.V (Guadalajara, Mexico), registered in the State Register of Veterinary Drugs and Feed Additives of the Ministry of Agriculture of the Republic of Kazakhstan, was used for comparison. The commercial vaccine contained at least 256 HA units (HAU) of recH5 recombinant antigen (strain A/duck/China/E319-2/03, subtype H5N1, clade 2.3.2) and 128 HA units of NDV antigen (strain LaSota) per dose (0.5 mL), obtained in ECEs. Both viral antigens (AI+ND) were produced as an oil-based adjuvant vaccine (70:30 by weight).

**Table 1:** Vaccine formulations and routes of administration.

Group	Description of the adjuvant/vaccine/controls	Antigen HAU in HA assay	Adjuvant to antigen ratio, wt.%	Method of administration
1	Montanide ISA 78 VG + antigen*	128	70:30	SC
2	Montanide ISA 71 R VG + antigen*	128	70:30	SC
3	Montanide GEL P PR + antigen*	128	10:90	SC
4	Commercial vaccine VOLVAC^®^ B.E.S.T AI+ND (oil-based adjuvant + antigen**)	256	70:30	SC
5	PBS + antigen*	128	70:30***	SC
6	Mannose-conjugated chitosan nanoparticles + antigen*	128	N/A	IN
7	Montanide GEL P PR + antigen*	128	10:90	IN
8	PBS + antigen*	128	10:90***	IN
9	PBS alone	–	–	SC, IN

HA, hemagglutination; HAU, hemagglutination units; IN, intranasal; SC, subcutaneous; PBS, phosphate-buffered saline; N/A, not applicable.

*Candidate vaccine strain IDCDC-RG71A (H5N8) inactivated with 0.1% formaldehyde.

**Recombinant baculovirus recH5 encoding HA of the A/dk/China/E319-2/2003 (H5N1) strain of HPAI virus belonging to clade 2.3.2 (Genbank accession AY518362.1).

***PBS to antigen ratio.

The detailed information on preparing the vaccine formulations is given in **Table 1**.

#### 2.4.1 Vaccine formulations with Montanide ISA 78 VG or Montanide ISA 71 R VG adjuvants

To prepare the experimental vaccine formulations (groups 1 and 2), adjuvants Montanide ISA 78 VG and Montanide ISA 71 R VG (Seppic, France) and vaccine candidate virus antigen (aqueous phase; 128 HAU) were mixed in a 70:30 ratio by wt.% by stirring at 4,000 rpm using an IKA Ultra Turrax^®^ Tube Drive Basic high shear stirrer (Ref. 3646000; IKA, Germany) with a DT-20 rotor–stator insert tube (Ref. 3703100; IKA, Germany) working with a volume of 2–15 mL. The adjuvant was placed in the DT-20 rotor–stator tube, and the aqueous phase was carefully added to the same tube without stirring, ensuring that the emulsion temperature was below 20°C before initiating the mixing process. Preemulsification step: The tube was connected to the docking station, and the mixing speed was set to 1,100 rpm (speed level “3”). The mixing process was then carried out for 2 min. Emulsification step: The stirring speed was set to 4,000 rpm (speed level “9”), and the mixing process was carried out for 10 min. The prepared formulations are poured into sterile 10-mL vials, sealed, and stored at 4°C until testing.

#### 2.4.2 Vaccine formulation with Montanide GEL P PR adjuvant

To prepare the experimental vaccine formulation (groups 3 and 7), the Montanide GEL P PR adjuvant and the candidate vaccine antigen (aqueous phase; 128 HAU) were mixed in a ratio of 10:90 by wt.% by stirring at 200 rpm using the Stegler HS-Pro DT magnetic stirrer (Stegler, China) for 5–10 min at RT. The prepared formulation is poured into 10-mL sterile vials, sealed, and stored at 4°C until testing.

#### 2.4.3 Antigen and negative control

After confirming complete inactivation, antigen, 128 HAU/dose in PBS, was diluted in ratios of 70:30 (group 5) and 10:90 (group 8). PBS alone (group 9) was used as a negative control.

#### 2.4.4 Vaccine formulation with mannose-conjugated chitosan nanoparticles

The vaccine antigen (128 HAU) and mannose-conjugated chitosan nanoparticles (mCS NPs) formulation (group 6) was prepared by using a standard ionic gelation method as described previously (22). The mCS NPs morphology, antigen loading efficiency, and size distribution were determined using appropriate methods. The vaccine formulation was lyophilized and stored at −20°C until use. Resuspension of the mCS NP-vaccine was carried out with PBS to the desired volume. All vaccine formulations were kept sterile and contained <2 EU/dose endotoxin.

### 2.5 Safety, immunogenicity, and efficacy of experimental HPAI (clade 2.3.4.4b) vaccines administered via parenteral and mucosal routes in a single and double immunization regimen in chickens

Ninety White Leghorn pullets, negative for specific antibodies (SAN), were used to assess the safety, immunogenicity, and efficacy of the experimental HPAI (clade 2.3.4.4b) vaccines administered via parenteral or mucosal routes in a single and double immunization regimen in chickens according to the study design (**Table 2**).

**Table 2:** Study design of safety, immunogenicity, and efficacy of the experimental HPAI (clade 2.3.4.4b) vaccines via parenteral and mucosal routes in a single and double immunization regimen in chickens.

Vaccine Antigen: RG-H5N8 Adjuvant: ISA-78	Vaccine Antigen: RG-H5N8 Adjuvant: ISA-71-R	Vaccine Antigen: RG-H5N8 Adjuvant: GEL-P	Vaccine Antigen: recH5 Adjuvant: Oil-based	Vaccine Antigen: RG-H5N8 Adjuvant: None	Nanovaccine Antigen: RG-H5N8 Adjuvant: mCS-NPs*	Vaccine Antigen: RG-H5N8 Adjuvant: GEL-P*	Vaccine Antigen: RG-H5N8 Adjuvant: None	Negative control: PBS alone
Group 1	Group 2	Group 3	Group 4	Group 5	Group 6*	Group 7*	Group 8*	Group 9
Volume of vaccine or negative control administered								
0.5 mL	0.5 mL	0.5 mL	0.5 mL	0.5 mL	0.5 mL	0.5 mL	0.5 mL	0.5 mL
Single or double immunization schedule								
SC	SC	SC	SC	SC	IN*	IN*	IN*	SC and IN**
Number of pullets per group (Leghorn)								
N = 10	N = 10	N = 10	N = 10	N = 10	N = 10	N = 10	N = 10	N = 10
A 35-day clinical follow-up with weekly weight monitoring								
N = 10	N = 10	N = 10	N = 10	N = 10	N = 10	N = 10	N = 10	N = 10
Blood tests for IgY, IgA, and anti-hemagglutinin antibodies 0, 7, 14, 21, and 28 days after vaccination (immunogenicity)								
N = 10	N = 10	N = 10	N = 10	N = 10	N = 10	N = 10	N = 10	N = 10
Intranasal challenge of pullets with virulent strain A/mute swan/Mangystau/1-S24R-2/2024 (H5N1; clade 2.3.4.4b) at 35 days after vaccination and 10 days of clinical observation with daily body temperature monitoring								
N = 5	N = 5	N = 5	N = 5	N = 5	N = 5	N = 5	N = 5	N = 5
Collection of tracheal and cloacal swabs on days 2, 4, and 6 after challenge								
N = 5	N = 5	N = 5	N = 5	N = 5	N = 5	N = 5	N = 5	N = 5
Euthanasia and collection of respiratory, intestinal, lymphoreticular, and nervous system tissues for histologic studies 10 days after challenge								
N = 5	N = 5	N = 5	N = 5	N = 5	N = 5	N = 5	N = 5	N = 5

PBS, phosphate-buffered saline; SC, subcutaneous; IN, intranasal; RG-H5N8, reverse genetics, candidate vaccine strain IDCDC-RG71A (H5N8) inactivated with 0.1% formaldehyde; mCS-NPs, mannose-conjugated chitosan nanoparticles; ISA-78 VG, Montanide ISA 78 VG; ISA-71-R, Montanide ISA 71 R VG; GEL-P, Montanide GEL P PR; recH5, recombinant baculovirus recH5 encoding HA of the A/dk/China/E319-2/2003 (H5N1) strain of HPAI virus belonging to clade 2.3.2 (Genbank accession AY518362.1); IgY, Immunoglobulin Y.

*Vaccine was administered in a double regimen intranasally with an interval of 14 days.

**Administration of PBS via two routes (SC and IN) at the same time.

The pullets at the age of 4 weeks were divided into nine experimental groups by randomization with 10 White Leghorns in each group on single (groups 1–5) and double (groups 6–8) immunization regimens. In groups 1–3, experimental vaccine formulations containing ISA-78, ISA-71-R, and GEL-P adjuvants were administered subcutaneously; in group 4, a commercial-oil-adjuvanted recH5 vaccine was administered subcutaneously for comparison; in groups 6 and 7, the vaccine with mCS-NPs and GEL-P adjuvant was administered intranasally; in groups 5 and 8, the vaccine without adjuvant was administered subcutaneously and intranasally, respectively; and in group 9 (negative control), PBS was administered instead of the vaccine, both subcutaneously and intranasally simultaneously.

#### 2.5.1 Sample collection

Blood samples were collected from the wing vein for antibody analysis at 7, 14, 21, and 28 days post-vaccination in the vaccinated and control groups. After blood clotting, the samples were centrifuged at 5,000 × *g* for 10 min at 4°C to collect serum, which was stored in aliquots at −80°C until tested. The tracheal and cloacal swab samples were collected at 2, 4, and 6 days post-challenge in all groups (**Table 2**). The swab samples were resuspended in 1 mL of DMEM (Gibco, UK) supplemented with 2,000 mg/mL streptomycin and 2,000 IU/mL penicillin. The suspensions were centrifuged at 3,000 × *g* for 10 min, and 0.2 mL of the supernatants from the tracheal or cloacal swabs was used to inoculate the MDCK cells.

#### 2.5.2 Safety

The chickens were examined daily for 35 days for clinical symptoms (activity, appetite, and respiratory and digestive status) and the presence or absence of other abnormalities. All chickens were weighed weekly to monitor their growth dynamics.

#### 2.5.3 Immunogenicity

The immunogenicity of the experimental vaccine in chickens was determined by indirect enzyme-linked immunosorbent assay (iELISA) to measure influenza-specific immunoglobulin Y (IgY) and immunoglobulin A (IgA) antibody levels and by hemagglutination inhibition (HI) assay. Peripheral blood mononuclear cells (PBMCs) were used for the analysis of CD4 + and CD8 + T cell proliferation.

Detection of anti-H5 influenza IgY or IgA antibodies was performed by iELISA using Jet Biofil plates (#FEP-101-896; Guangzhou, China) precoated overnight at 2°C–8°C with inactivated candidate vaccine strain RG71A (128 HAU) diluted 1:10 in a commercial coating buffer (#B288159, BioLegend). The coated plates were blocked using an ELISA Assay Diluent (#421203, BioLegend) at 200 μL per well and incubated under constant shaking (300–330 rpm on a PST-60HL thermal shaker, Biosan) for 1 h at RT. Sera from vaccinated and non-vaccinated chickens were individually titrated (IgY only) twofold from dilutions 1:500–1:2,048,000, and 100-μL samples were added from each dilution to the wells and incubated under constant shaking (300–330 rpm) for 1.5–2 h at RT. After washing (×4), secondary goat anti-chicken IgY H&L (HRP; 1:50,000, #ab6877, Abcam, MA, USA) or goat anti-chicken IgA H&L (HRP; 1:10,000, #ab112817, Abcam, MA, USA) antibodies were added, and the plates were incubated (1 h at RT with shaking). After additional washing (×4), the plates were incubated with streptavidin–HRP conjugate [1:10,000 final dilution for IgY or 1:20,000 final dilution for IgA (Pierce #21130)] for 30 min at RT with shaking. Finally, the plates were washed (five times) and added with a ready-to-use TMB substrate (#N301, Thermo Fisher Scientific, 100 μL per well). The color reaction was stopped by adding a stop solution (#B308260, BioLegend, 100 μL per well), and the optical density (OD) was measured (measuring wavelength 450 nm, reference wavelength 630 nm) on a ChroMate 4300 analyzer (Awareness Technology, Inc.). The cutoff value for determining seropositivity was the average OD value of the negative sample + three times the standard deviation (23).

The HI assay was performed using the following standard protocol (24). Briefly, cholera filtrate was used as a receptor-destroying enzyme (RDE) according to the WHO protocol (25) to remove innate inhibitors from the serum that could interfere with the assay. The serum was then heated to 56°C for 30 min to remove nonspecific hemagglutination inhibition factors and to inactivate the cholera filtrate. The RDE-treated serum samples (25 µL) were diluted twofold with PBS (25 µL) in 96-well V-bottom plates and incubated with 4 HA units (HAU) of the candidate vaccine strain RG71A for 30 min at RT. Then, 50 µL of a 1% suspension of RBCs was added to each well and incubated at RT for 30 min for the readout. The HI titer was expressed as the reciprocal (log_2_ titers) of the highest serum dilution that completely inhibited hemagglutination. Serum HI titers equal to or >1:16 (>4 log_2_) were considered positive, while sera with titers in between 1:10 and 1:16 (3.3 to 4 log_2_) or with undetectable antibodies were considered negative. The limit of detection was at dilution 1:5 (2.3 log_2_), and samples with undetectable titers were assigned a dilution value of 1:5 (2.3 log_2_) for statistical purposes.

PBMCs were isolated from chicken blood. Approximately 3 mL of peripheral blood was collected from the subclavian vein of each bird following a sterile procedure and immediately transferred to tubes containing EDTA. The leukothrombocytic layer (750 µL) was separated by centrifugation at 1,800 rpm for 40 min. Subsequently, 750 µL of Histopaque R-1077 was placed into a 2-mL microcentrifuge tube, and 750 µL of the leukothrombocytic layer was gently layered on top of it. Centrifugation was performed for 30 min at 1,800 rpm at RT. Mononuclear cells were aspirated from the opaque surface of the top layer. The cells were washed three times with sterile PBS and then once with RPMI 1640 medium by centrifugation at 1,000 rpm for 10 min. The cells were resuspended in 1 mL of RPMI 1640 and counted using an automated cell counter (Countess II FL; Thermo Fisher Scientific Inc.). The PBMCs were used for lymphocyte proliferation assay study. The CellTrace™ Violet Cell Proliferation Kit for flow cytometry (Thermo Fisher Scientific) was used according to the manufacturer's instructions. Labeled lymphocytes were cultured for 5 days at 37 °C in 5% CO2, both in the presence of the vaccine antigen at a HA titer of 1:128 and in its absence. Flow cytometry analysis of PBMCs from post-immunized chickens was performed using monoclonal antibodies (MAbs) against chicken CD8 alpha (CT-8), PECyanine5 (#MA528727, Invitrogen, USA), and CD4 (CT-4), fluorescein isothiocyanate (FITC; #MA528685, Invitrogen, USA). Briefly, 100 μL of PBMCs (10^5^–10^6^ cells) in PBS was mixed with 5 μL of MAbs (0.5 μg/μL) in separate tubes, each with an isotype control for individual birds. The cells were gently mixed and incubated at 37°C for 1 h. The tubes were then washed three times with a washing/blocking buffer containing PBS, 1% bovine serum albumin (BSA), and 0.1% sodium azide (SA) and centrifuged at 4,000 rpm for 5 min. The cells were then resuspended, fixed with 0.5% paraformaldehyde (PFA) for 30 min at RT, and analyzed on an Attune™ NxT Flow Cytometer (Thermo Fisher Scientific, USA) using Attune NxT Software (Thermo Fisher Scientific, USA). Analysis of lymphocyte proliferation was performed as previously described by us (26) in the FCS/SSC dot-plot lymphocyte isolate. CD4+ and CD8+ T-cell frequency was calculated as the difference (Δ) between antigen-stimulated and unstimulated samples from the total number of live proliferating lymphocytes and expressed as a percentage.

#### 2.5.4 Efficacy

To test the efficacy of the vaccine formulations at 35 days after immunization, five chickens from each group were transferred to the ABSL-3 facility and challenged with a dose of 10^6^ EID_50_ (embryonic infectious dose 50%) of the A/mute swan/Mangystau/1-S24R-2/2024 (H5N1) strain of HPAI in a volume of 500 µL by the intranasal route. Virus back-titration was performed in ECEs immediately following inoculation, confirming that birds received 10^6^ EID_50_. The birds were then monitored daily for 10 days for clinical signs and mortality with daily weighing and body temperature measurement. We considered rectal temperatures greater than or equal to 42.3°C as abnormally high temperatures based on this threshold for potential disease severity (27). The efficacy (protection) of the vaccine was calculated according to the following equation (6):


$$\text{Protection }\left(\%\right)\;=\;\frac{\text{Number of survivors}}{\text{Total number of challenged birds}}\;\;\times 100$$

Tracheal and cloacal swabs were collected from each bird on days 2, 4, and 6 after challenge to measure the level of viral shedding across the different groups by calculating the tissue culture infectious dose 50% (TCID_50_) per 1 mL of swab sample in 96-well plates of MDCK cells. After incubation at 37°C for 120 h, the plates were observed daily for the presence of cytopathic effect (CPE) by means of an inverted optical microscope. Then, the cell supernatants were harvested and transferred to V-bottom 96-well plates. The presence of the virus was detected using a hemagglutination assay (16). The endpoint titers were calculated according to the Reed and Muench method (21) based on six replicates for titration. Virus titers are expressed as log_10_ TCID_50_/mL.

#### 2.5.5 Histological analysis

Dead and euthanized chickens after the challenge were necropsied, and tissues from the respiratory (lung, trachea), digestive (liver, pancreas), intestinal (large and small intestine), lymphoreticular (spleen and bursa of Fabricius), and nervous system (brain) were collected for histological studies. Necropsy and histopathological studies were evaluated as previously described (23, 28) using the following five-level histological scale: 0 (no changes; 0%), 1 (mild inflammation; <25%), 2 (moderate inflammation; 26%–50%), 3 (pronounced inflammation; 51%–75%), and 4 (severe inflammation; 76%–100%) (29). Briefly, the chicken tissues were fixed in 10% buffered formaldehyde, washed in water, and treated with four portions of 100% isopropyl alcohol and two portions of xylene. Subsequently, the tissues were soaked in four portions of paraffin to create paraffin blocks, which were then used to prepare 5-µm sections using a microprocessor-controlled microtome (MZP-01, KB Technom, Russia). The tissue sections were deparaffinized in two portions of xylene and three portions of ethyl alcohol with decreasing concentrations (96%, 80%, and 70%) and stained with hematoxylin (BioVitrum, Russia) and eosin (DiaPath, Italy). Following clarification in ascending ethyl alcohol concentrations (70%, 80%, and 96%) and two portions of xylene, the sections were covered with coverslips using Bio Mount synthetic medium (Bio Optica, Italy). The slides were observed under an Mshot microscope (model MF52-N, China), and photographs were taken at ×100 and ×400 magnification using an Mshot MS23 camera with the Mshot Image Analysis System program. Also, using this program, a measuring scale of 100 and 500 µm was placed on the photographs. A standardized scale was used for calibration, and all measurements were made in micrometers.

### 2.6 Statistical analysis

GraphPad Prism 9.0.0 (GraphPad Software, San Diego, CA, USA) was used for preparing graphs and for the statistical analysis of the experimental data. Differences in hematological parameters, antibody titers, viral load in swabs, and tissues between animal groups were assessed using Tukey’s multiple comparisons test or Dunnett’s multiple comparisons tests or Holm–Šídák’s multiple comparisons test. The detection limit of the infectivity titer was 0.7 log_10_ TCID_50_/mL. The detection limit of IgY titers was 1:500 (9.0 log_2_). The limit of detection of HI titers was at dilution 1:5 (2.3 log_2_), and samples with undetectable titers were assigned a dilution value of 1:5 (2.3 log_2_) for statistical purposes. For all comparisons, *P <*0.05 was considered a significant difference.

## 3 Results

### 3.1 Experimental vaccine preparation, safety, and potency

The candidate vaccine strain IDCDC-RG71A (H5N8; clade 2.3.4.4b) was propagated in embryonated chicken eggs, and the virus was harvested from allantoic fluids after 48 h. The fluid containing 128 HAU/50 μL was inactivated using 0.1% formaldehyde, with complete inactivation confirmed by the absence of viral growth in ECEs after three consecutive passages. Various vaccine formulations were prepared using different adjuvants: (1) ISA-78 and ISA-71-R mixed with the antigen at a 70:30 ratio, (2) GEL-P mixed at a 10:90 ratio, (3) mCS-NPs prepared using ionic gelation, lyophilized, and stored at −20°C, and (4) controls that included PBS alone and PBS with antigen at 70:30 and 10:90 ratios.

The safety of the experimental vaccines was evaluated by administering them to SAN chickens and monitoring for any adverse reactions over a 5-week period prior to challenge. The chickens were examined daily for 35 days for clinical signs including activity, appetite, and respiratory and digestive status as well as the presence or absence of abnormalities. All chickens were weighed weekly to monitor their growth dynamics (**Supplementary Table S1**). All vaccinated chickens, including those in groups ISA-78-SC, ISA-71-R-SC, GEL-P-SC, mCS-NPs-IN, and GEL-P-IN, showed no local or systemic reactions to the vaccine, with the vaccine formulations all being well tolerated (data not shown) in a similar manner to the commercial vaccine recH5-SC. In conclusion, the experimental vaccines all demonstrated effective inactivation, safety, and tolerance in SAN chickens, validating their potential as vaccine candidates for further evaluation.

### 3.2 Immunogenicity of the experimental and commercial vaccines in chickens

**Figure 1:**
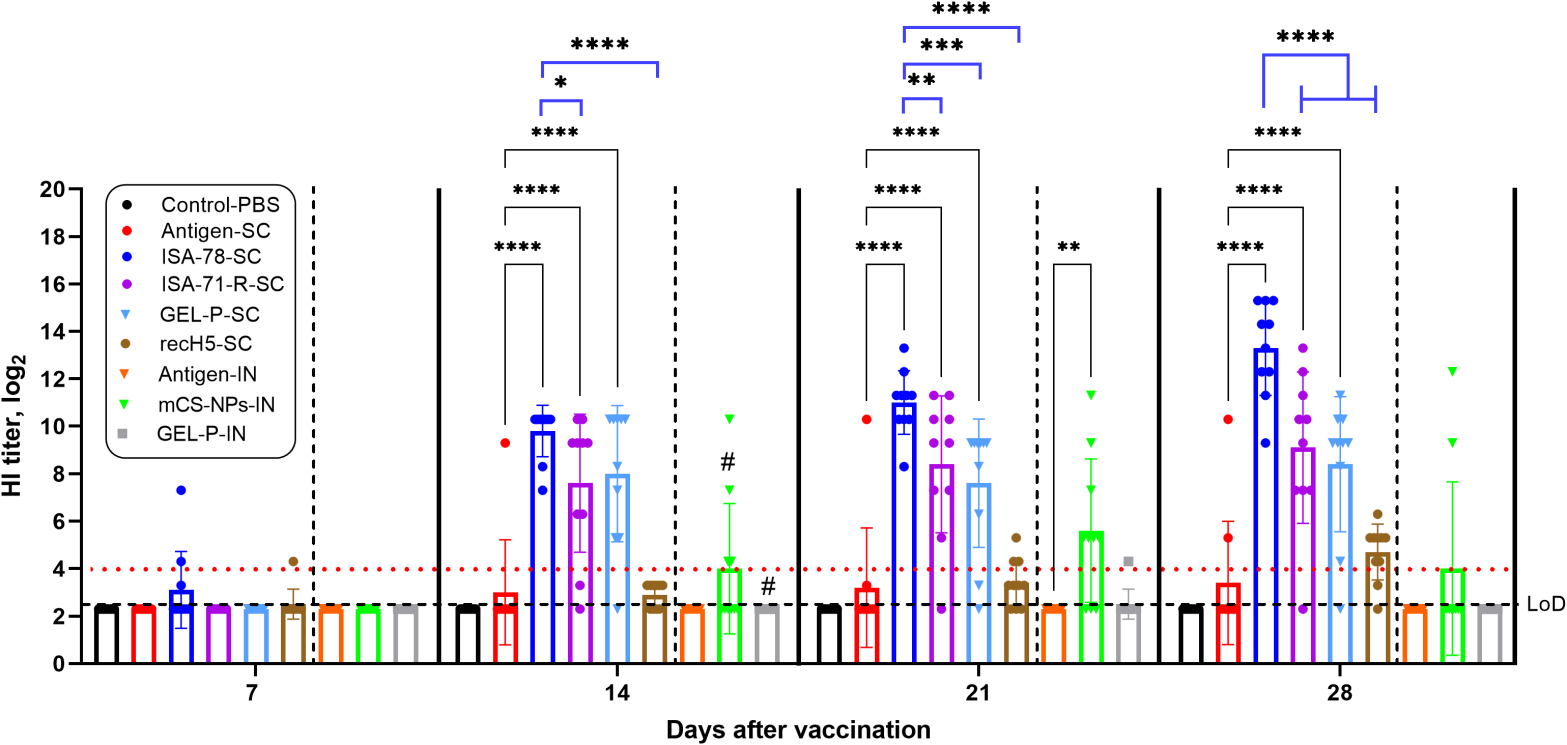
Detection of anti-hemagglutinin antibodies in chickens vaccinated with experimental and commercial recH5-SC vaccines by HI assay. Serum HI titers equal to or >4 log_2_ were considered positive (horizontal red dotted line), while sera with titers in between 3.3 and 4 log_2_ or with undetectable antibodies were considered negative. The limit of detection (LoD) was at dilution 2.3 log_2_ (horizontal dotted line). #, second intranasal vaccination for groups GEL-P-IN and mCS-NPs-IN. Data are mean ± SD titer of 10 chickens in each group. Statistical differences between groups were assessed using Tukey’s and Dunnett’s multiple comparisons tests. For all comparisons, *P* < 0.05 was considered a significant difference. *P = 0.0354; **P = 0.0010, and 0.0075; ***P = 0.0002; ****P < 0.0001.

The immunogenicity of the experimental and commercial vaccines in chickens was assessed by measuring HI antibody levels at 7, 14, 21, and 28 days post-immunization (**Figure 1**). By day 7, HI antibody responses were detected in groups ISA-78-SC and recH5-SC with seropositivity rates of 20% and 10%, respectively. By day 14, HI seropositivity reached 100% in ISA-78-SC, 90% in GEL-P-SC, 80% in ISA-71-R-SC, and 30% in mCS-NPs-IN. At day 21, seropositivity remained at 100% in group ISA-78-SC, while groups ISA-71-R-SC, GEL-P-SC, mCS-NPs-IN, recH5-SC, GEL-P-IN, and antigen-SC exhibited rates of 90%, 80%, 70%, 30%, 10%, and 10%, respectively. By day 28, group ISA-78-SC maintained 100% seropositivity, with groups GEL-P-SC, ISA-71-R-SC, recH5-SC, mCS-NPs-IN, and antigen-SC showing 90%, 90%, 80%, 20%, and 20% seropositivity, respectively.

On days 14, 21, and 28 post-vaccination, HI antibodies in groups ISA-78-SC, GEL-P-SC, and ISA-71-R-SC were significantly higher (*P* < 0.0001) compared to those in antigen-SC. In group mCS-NPs-IN, the HI antibody levels were significantly elevated (*P* = 0.0010) compared to the antigen-IN group only at 21 days post-vaccination. Group ISA-78-SC exhibited significantly higher HI antibody levels on days 21 and 28 post-vaccination compared to groups GEL-P-SC (*P* = 0.0002; *P* < 0.0001), ISA-71-R-SC (*P* = 0.0075; *P* < 0.0001), and recH5-SC (*P* < 0.0001). At day 14 post-vaccination, a similarly significant difference was observed only with groups ISA-71-R-SC (*P* = 0.0354) and recH5-SC (*P* < 0.0001).

Overall, the adjuvanted SC formulations GEL-P-SC and ISA-71-R-SC showed robust HI responses and were not significantly different among each other. However, the ISA-78-SC group demonstrated superior immunogenicity, achieving 100% seropositivity and significantly higher antibody levels compared to other SC groups. These findings suggest that ISA-78-SC formulation is more effective in inducing a strong and sustained immune response.

**Figure 2:**
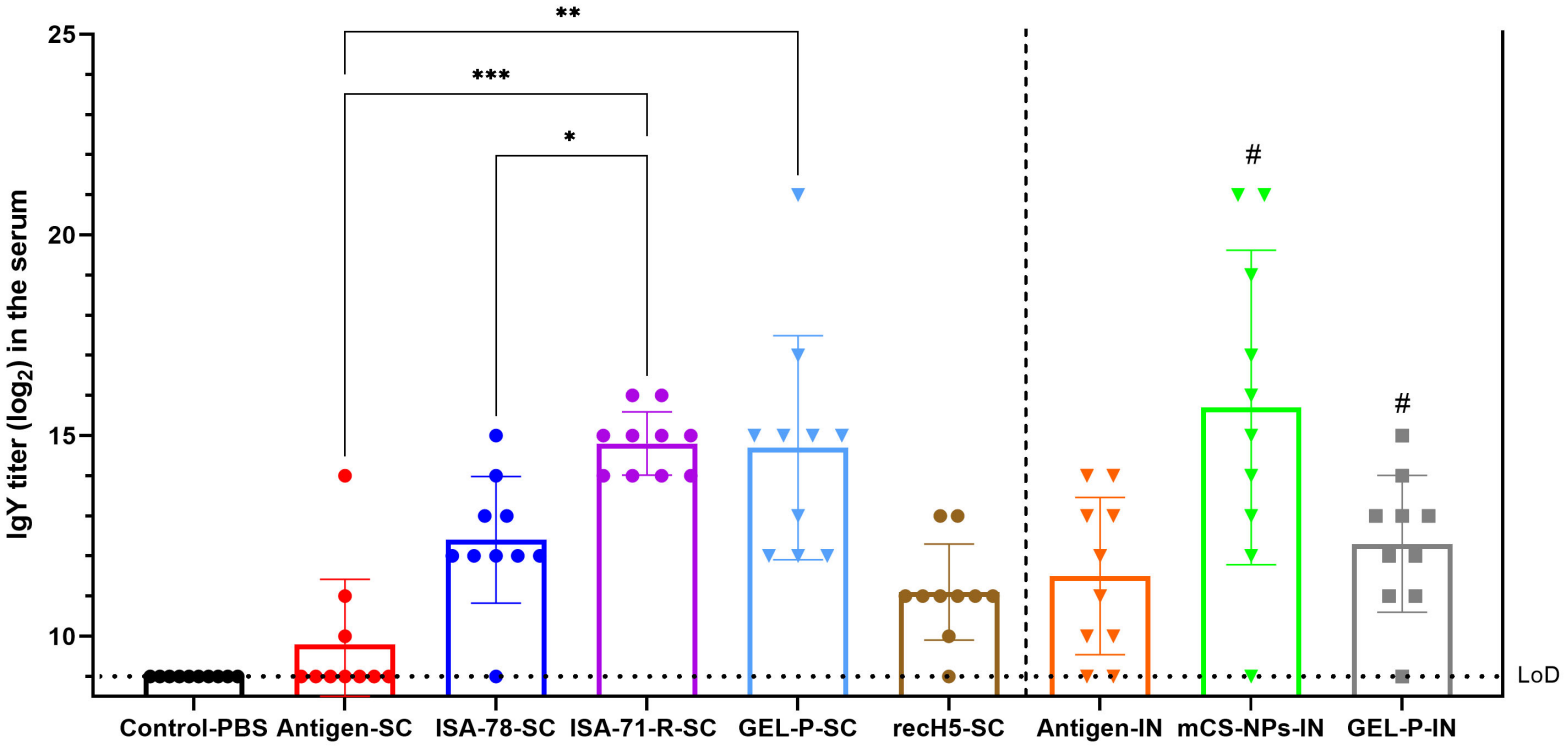
Detection of IgY antibodies in chickens vaccinated with experimental and commercial vaccines by iELISA. IgY is the primary immunoglobulin in birds, reptiles, and amphibians, functionally equivalent to mammalian IgG. It plays a critical role in humoral immune response by neutralizing pathogens and providing immunity. The limit of detection (LoD) was at titer 9.0 log_2_ (horizontal dotted line). #—A second intranasal immunization was administered to groups GEL-P-IN and mCS-NPs-IN at 14 days after prime vaccination. Data are mean ± SD titer of 10 chickens in each group. Statistical differences between groups were assessed using Tukey’s multiple comparisons test. For all comparisons, *P* < 0.05 was considered a significant difference. *P = 0.0274; **P = 0.0015; ***P = 0.0008.

The immunogenicity of both experimental and commercial vaccines in chickens was evaluated by measuring the levels of serum immunoglobulin Y (IgY) 28 days after immunization (**Figure 2**). IgY is the primary serum antibody in birds, functioning similarly to mammalian IgG, and serves as a key marker of vaccine-induced immunity in avian models. Groups ISA-71-RS-C and Gel-P-SC showed the highest anti-influenza IgY levels, followed by group ISA-78-SC, which had intermediate levels and not significantly different to the commercial recH5-SC vaccine, and interestingly it was significantly lower (*P* = 0.0274) compared to ISA-71-R-SC. While most chickens that received antigen alone via SC route did not induce any IgY response, most of those that received antigen alone via IN route made serum IgY responses equivalent to the high and intermediate IgY responses of SC groups Gel-P-SC and ISA-78-SC, respectively. No significant difference in IgY was observed when comparing the mCS-NPs-IN and GEL-P-IN groups with the antigen-IN group.

**Figure 3:**
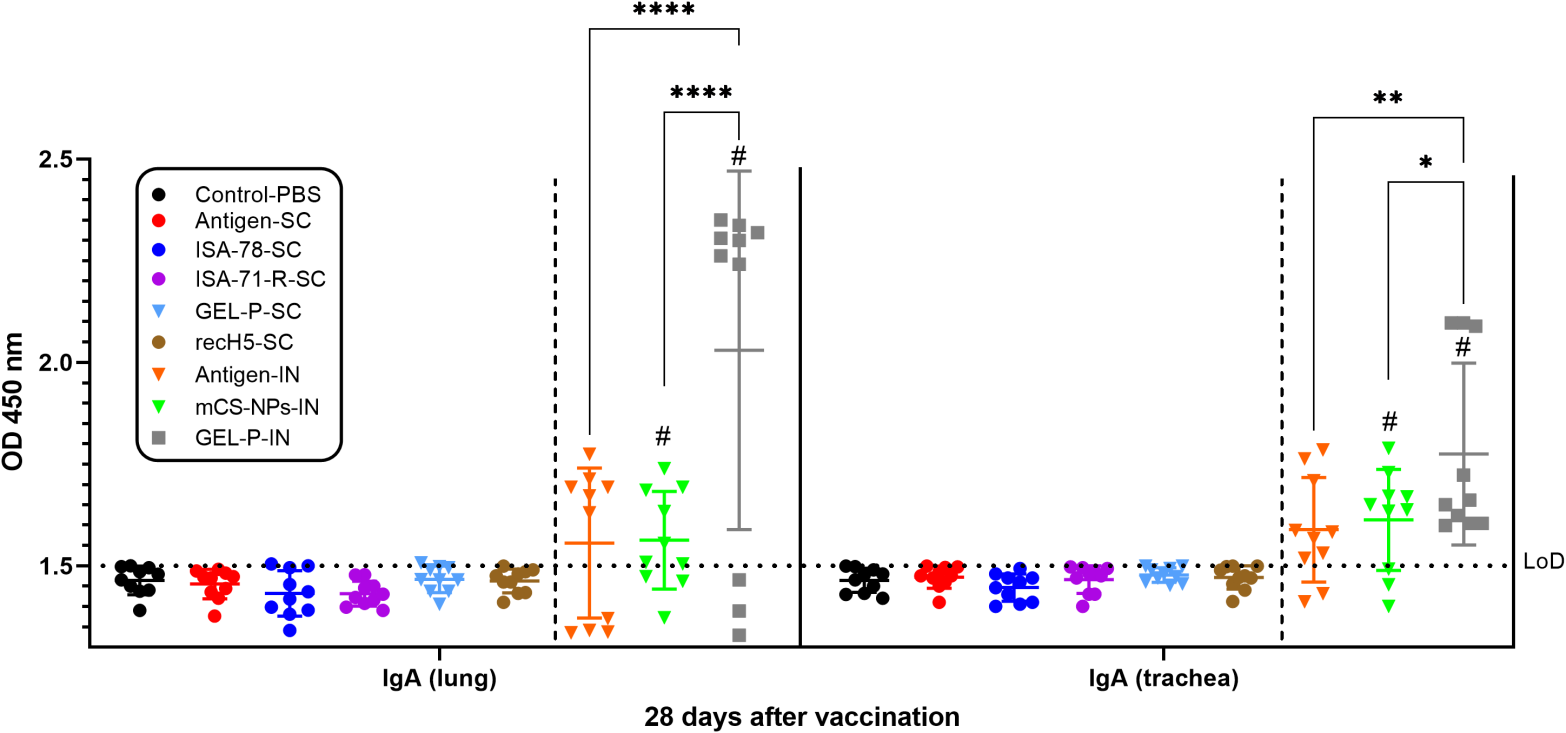
Detection of IgA antibodies in chickens vaccinated with experimental and commercial vaccine formulations by iELISA. The limit of detection (LoD) was 1.5 at an optical density (OD) with a wavelength of 450 nm (horizontal dotted line). ^#^A second intranasal immunization was administered to groups GEL-P-IN and mCS-NPs-IN at 14 days after prime vaccination. Data are presented as mean ± SD titers from 10 chickens in each group. Statistical differences between groups were assessed using Šídák’s multiple-comparison test. For all comparisons, *P* < 0.05 was considered a significant difference. *P = 0.0144; **P = 0.0019; ****P < 0.0001.

Immunogenicity was further assessed using iELISA by measuring influenza-binding IgA levels in lung and trachea on day 28 post-vaccination (**Figure 3**). Influenza-specific IgA in the lung and trachea was only detected in the IN groups (antigen-IN, and mCS-NPs-IN, and GEL-P-IN), with high influenza-binding IgA levels only seen in group GEL-P-IN.

**Figure 4:**
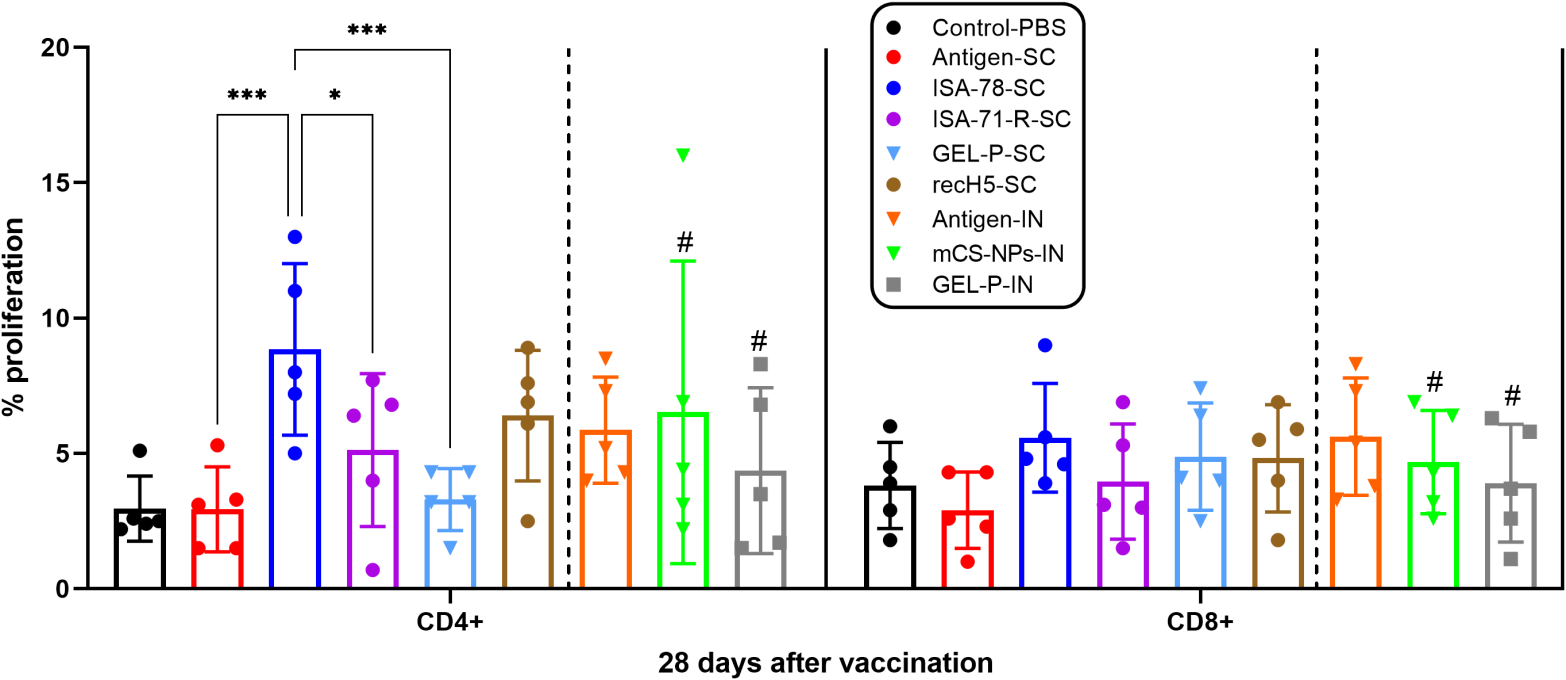
Antigen-stimulated CD4 + and CD8 + T cell proliferation in PBMCs from chickens vaccinated with experimental and commercial vaccines. ^#^A second intranasal immunization was administered to groups GEL-P-IN and mCS-NPs-IN at 14 days after prime vaccination. CD4+ and CD8+ T cell proliferation was calculated as the difference (Δ) in the number of proliferating lymphocytes between stimulated vs. non-stimulated cells. Data are mean ± SD titer of five chickens in each group. Statistical differences between groups were assessed using the Tukey’s multiple comparisons test. For all comparisons, *P* < 0.05 was considered a significant difference. *P = 0.0420; ***P = 0.0001 and *P* = 0.0003.

We assessed antigen-stimulated CD4+ and CD8+ T cell proliferation in PBMCs from chickens vaccinated with both experimental and commercial vaccines on day 28 post-vaccination (**Figure 4**). Only in group ISA-78-SC were the levels of CD4+ T cell proliferation significantly higher (*P* = 0.0001) compared to the SC antigen alone group. In addition, group ISA-78-SC showed significantly higher CD4+ T cell proliferation compared to groups ISA-71-RS-SC (*P* = 0.0420) and Gel-P-SC (*P* = 0.0003). There were no significant differences in CD8+ T cell proliferation levels between groups. Overall, ISA-78-SC exhibited the highest CD4+ T cell response.

### 3.3 Efficacy of the experimental and commercial vaccines in chickens

**Figure 5:**
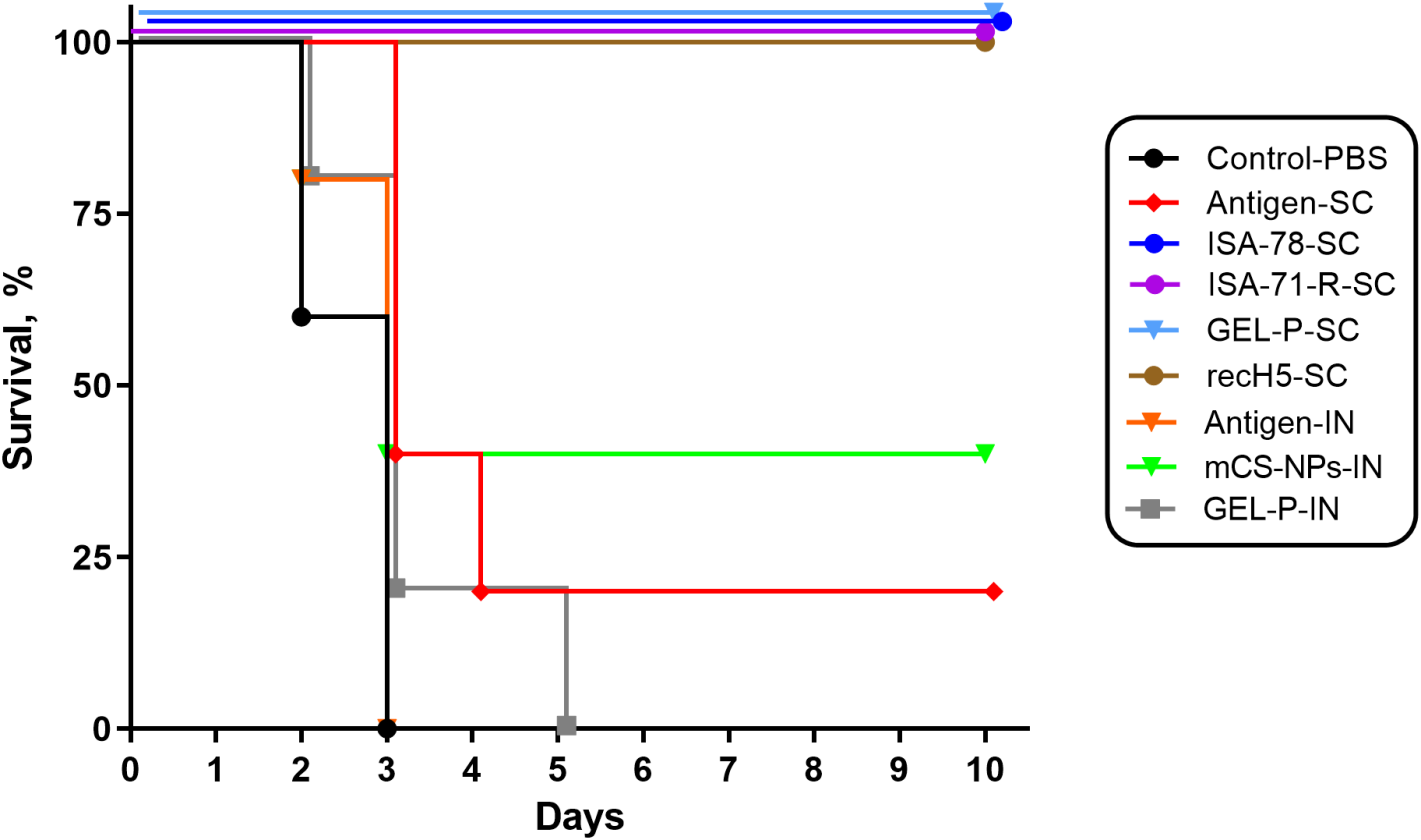
Kaplan–Meier survival curve of vaccinated and control chickens.

To assess the efficacy of vaccine formulations, chickens at 35 days post-immunization were transferred to our ABSL-3 facility and challenged with 10^6^ EID_50_ of the A/mute swan/Mangystau/1-S24R-2/2024 (H5N1) strain of HPAI via the IN route. The birds were monitored for 10 days for clinical signs (rectal temperature) and mortality, and efficacy was calculated based on survival rates (**Supplementary Table S2**; **Figure 5**).

High efficacy group (100% protection): The SC immunization groups, ISA-78-SC, ISA-71-R-SC, GEL-P-SC, and recH5-SC all demonstrated 100% protection. Moderate efficacy group (40% protection): The mCS-NPs-IN group exhibited 40% protection, consistent with the overall poor systemic immunogenicity responses in all of the IN groups despite receiving two vaccine doses. Low efficacy group (20% protection): The antigen alone SC group administered subcutaneously showed 20% protection, indicating that the SC route of vaccination provided better protection than the IN route. No efficacy group (0% protection): The IN groups GEL-P-IN and antigen-IN showed no protection, with all birds dying after the challenge. As expected, the control group, which received PBS via both subcutaneous and intranasal routes, exhibited 0% protection, with all birds dying after the challenge.

**Figure 6:**
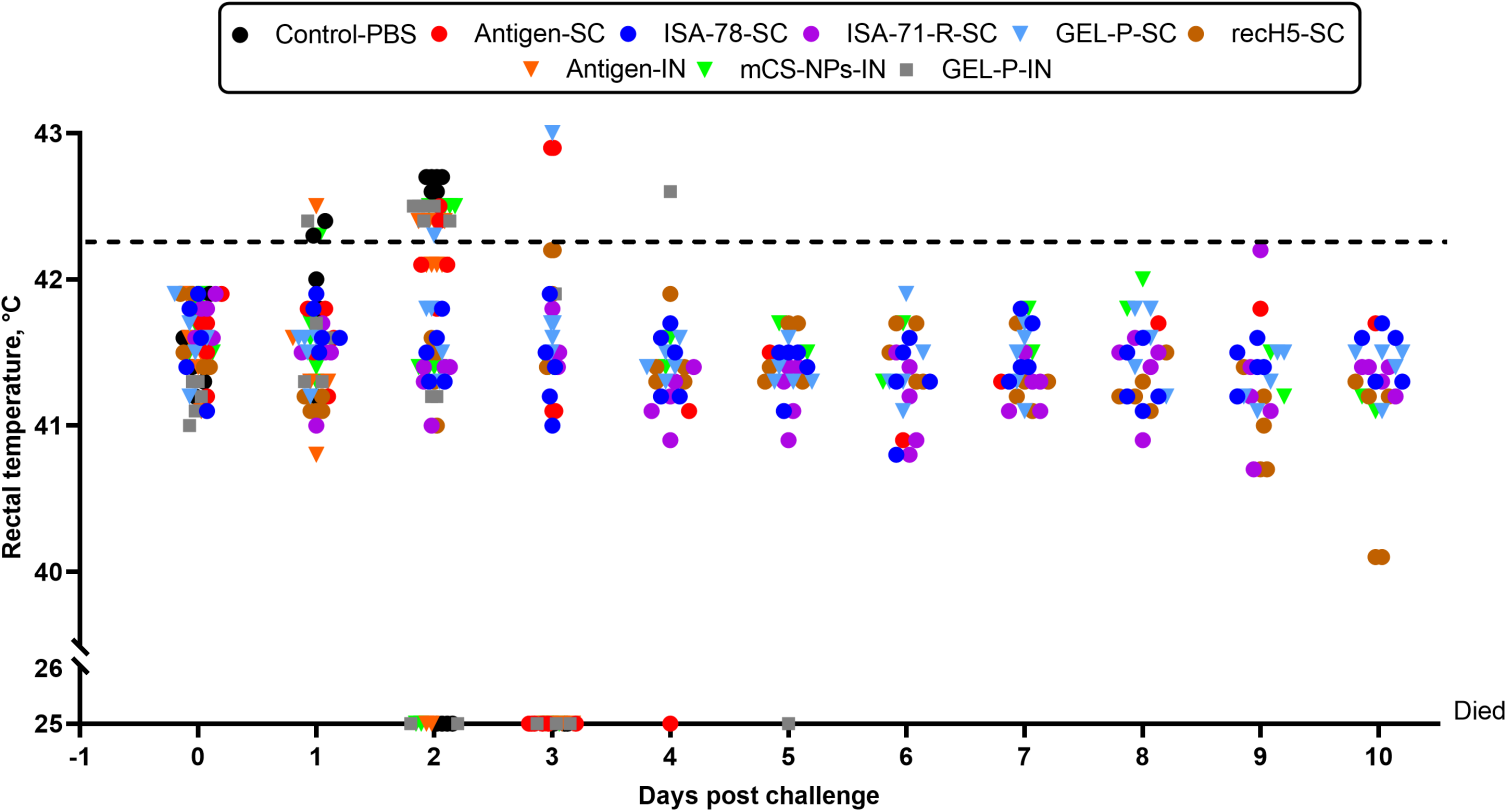
Daily body temperatures (°C) of chickens post-challenge. The horizontal dotted line indicates an abnormally high temperature (≥42.3°C) for White Leghorns.

The body temperature of the chickens was recorded daily for 10 days following the H5N1 challenge (**Supplementary Table S3**; **Figure 6**). High efficacy group: Groups ISA-78-SC, ISA-71-R-SC, GEL-P-SC, and recH5-SC had high temperature incidences, ranging from 0% (ISA-78-SC, ISA-71-R-SC, and recH5-SC) to 20% (GEL-P-SC). Despite some abnormally high temperature occurrences, all of these groups achieved 100% protection, showing that these vaccines prevented mortality even when some chickens still exhibited a high temperature in response to the infection.

Moderate and low efficacy groups: The moderate efficacy group (40% protection) and the low efficacy group (20% protection) both exhibited an abnormally high temperature incidence of 60%. This suggests that, while the mCS-NPs-IN formulation provides some immune response, it may not be potent enough to offer substantial protection, and the antigen-SC formulation appears relatively ineffective. In both cases, the observed febrile reactions may indicate poor immunogenicity or an insufficiently balanced immune response, failing to provide robust protection while still inducing temperature elevations in some individuals.

No efficacy group: Groups control-PBS and GEL-P-IN provided no protection and had the highest abnormally high temperature incidence (100%), whereas group antigen-IN also showed an abnormally high temperature incidence (60%).

This categorization highlights the relationship between protective efficacy and an abnormally high temperature incidence, with higher protection groups generally showing a lower abnormally high temperature incidence, while groups with minimal to no protection have higher temperature rates. This suggests that optimal adjuvant and delivery strategies are key to achieving high protection and low adverse reactions.

**Figure 7:**
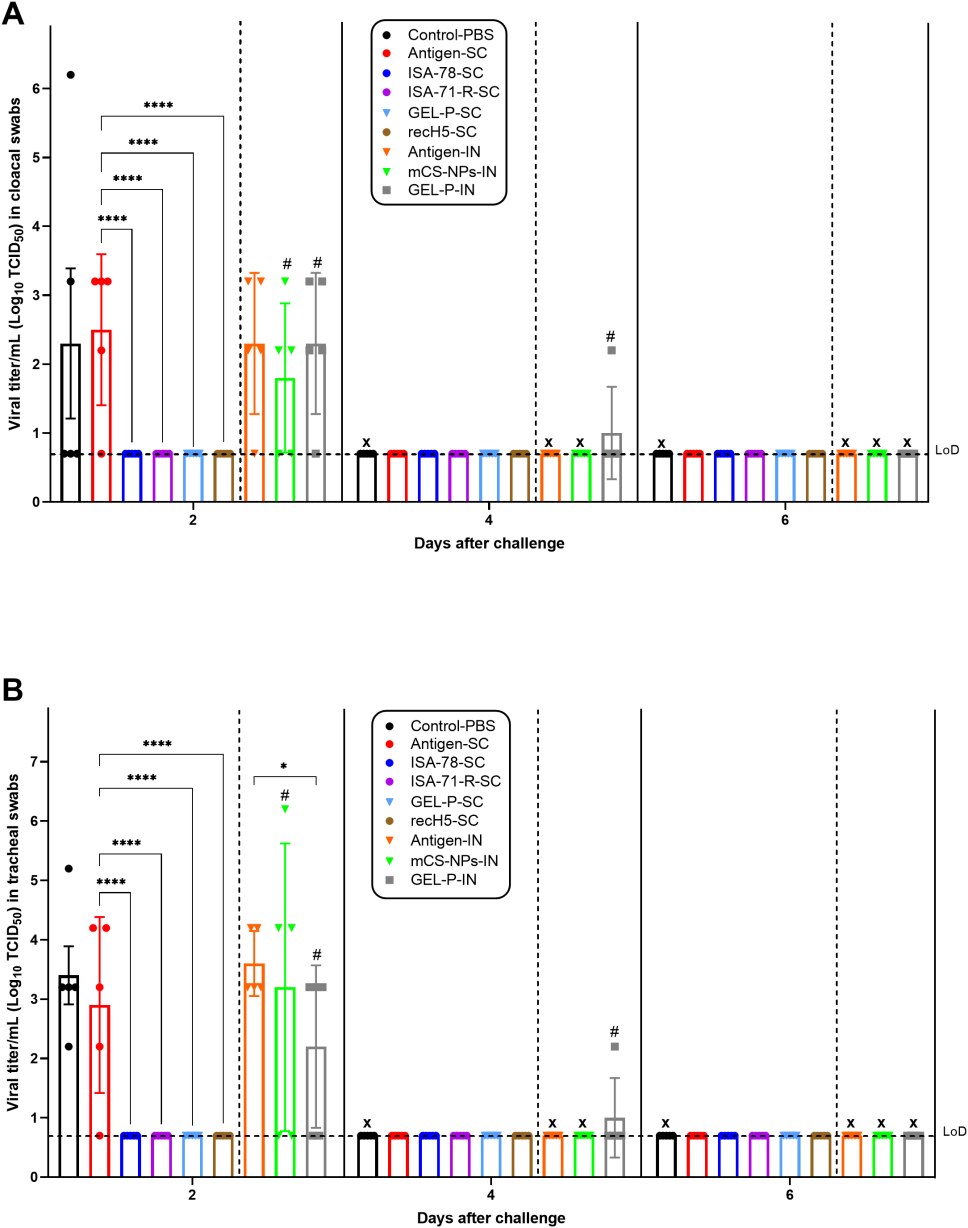
Viral shedding of the immunized chickens at 2, 4, and 6 days post-challenge. **(A)** Cloacal swabs and **(B)** tracheal swabs. The immunized chickens with experimental and commercial vaccine formulations were subjected to a challenge with 10^6^ EID_50_ of the A/mute swan/Mangystau/1-S24R-2/2024 (H5N1) strain of HPAI via the intranasal route. The limit of detection (LoD) was at titer 0.7 log_10_ TCID_50_ (horizontal dotted line). X, dead chickens. In graphs A and B, in the antigen-SC group at 4 days post-challenge, the X sign was omitted because one bird in this group remained alive until 10 days post-infection. Data are mean ± SD titer of five chickens in each group. Statistical differences between groups were assessed using Tukey’s multiple comparisons test. For all comparisons, *P* < 0.05 was considered a significant difference. *P = 0.0142; ****P < 0.0001. A second intranasal immunization was administered to groups GEL-P-IN and mCS-NPs-IN at 14 days after prime vaccination.

To investigate the capability of different vaccine formulations to control viral shedding after challenge with the A/mute swan/Mangystau/1-S24R-2/2024 (H5N1) strain, virus shedding in cloacal (**Figure 7A**) and tracheal (**Figure 7B**) swabs was measured at days 2, 4, and 6 post-infection. Adjuvanted SC groups ISA-78-SC, ISA-71-R-SC, GEL-P-SC, and recH5-SC demonstrated undetectable viral titers in cloacal and tracheal swabs on days 2, 4, and 6 post-challenge, indicating effective protection against viral shedding. In contrast, groups antigen-IN, mCS-NPs-IN, and GEL-P-IN, which received IN vaccines, as well as groups control-PBS and antigen-SC, exhibited viral titers in both cloacal and tracheal swabs on day 2 post-challenge. In the GEL-P-IN group, compared with the antigen-IN group, the viral titer in the trachea was lower on day 2 post-challenge; however, it remained detectable up to day 4 following the infection. This shows that SC vaccines were more effective in reducing viral replication and shedding compared to IN vaccines.

### 3.4 Histological analysis

The ability of the vaccines to prevent lung and liver tissue lesions caused by H5N1 virus infection was evaluated by a histological analysis of dead and euthanized chickens. It is worth noting that histological examination revealed no pathological changes in the trachea, pancreas, large and small intestine, spleen, bursa of Fabricius, or brain of the challenged chickens (data not shown).

**Figure 8:**
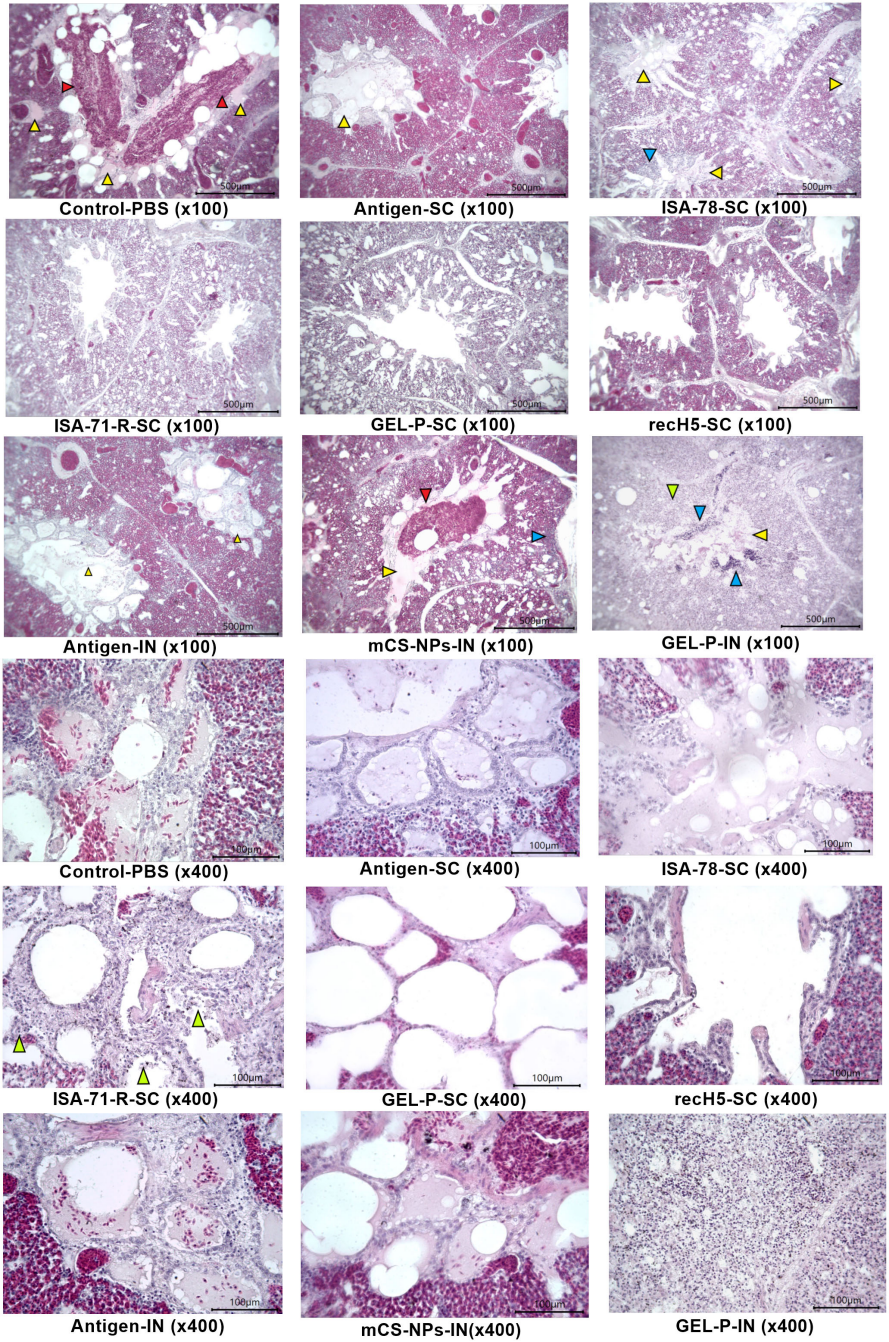
Histological analysis of lung tissue. Control-PBS (×100): transudate (yellow arrow) and mass accumulation of erythrocytes (red arrow) in the parabronchus cavity; antigen-SC (×100): transudate in parabronchus cavity (yellow arrow), erythrostasis in vessels; ISA-78-SC (×100): edematous fluid in the lumen of parabronchi (yellow arrows) and lymphocytic infiltrate in the atrial wall [blue arrow; ISA-71-R-SC (×100)]: parabronchial structure; GEL-P-SC (×100): structure of parabronchi; recH5-SC (x100): structure of parabronchi; antigen-IN (×100): transudate in parabronchus cavity (yellow arrow), erythrostasis in vessels; mCS-NPs-IN (×100): transudate (yellow arrow) and erythrocyte accumulation (red arrow) in the parabronchus cavity, as well as lymphocytic infiltrate in the walls of atria (blue arrow); GEL-P-IN (×100): necrosis of atria (green arrow), lymphocytic infiltration (blue arrow), and transudate in the parabronchus cavity (yellow arrow); control-PBS (×400): atria filled with transudate and erythrocytes, necrosis of atrial wall epithelium; antigen-SC (×400): atria filled with transudate and necrosis of atrial wall epithelium. ISA-78-SC (×400): transudate in atrial cavities and necrotized atrial walls; ISA-71-R-SC (×400): weak necrosis of atria epithelium (green arrow); GEL-P-SC (×400): structure of the atria; recH5-SC (×400): structure of the atria; antigen-IN (×400): atria filled with transudate and necrosis of atrial wall epithelium; mCS-NPs-IN (×400): atria filled with transudate and erythrocytes, necrosis of atrial wall epithelium; GEL-P-IN (×400): massive necrosis of atria; hematoxylin–eosin staining.

**Figure 9:**
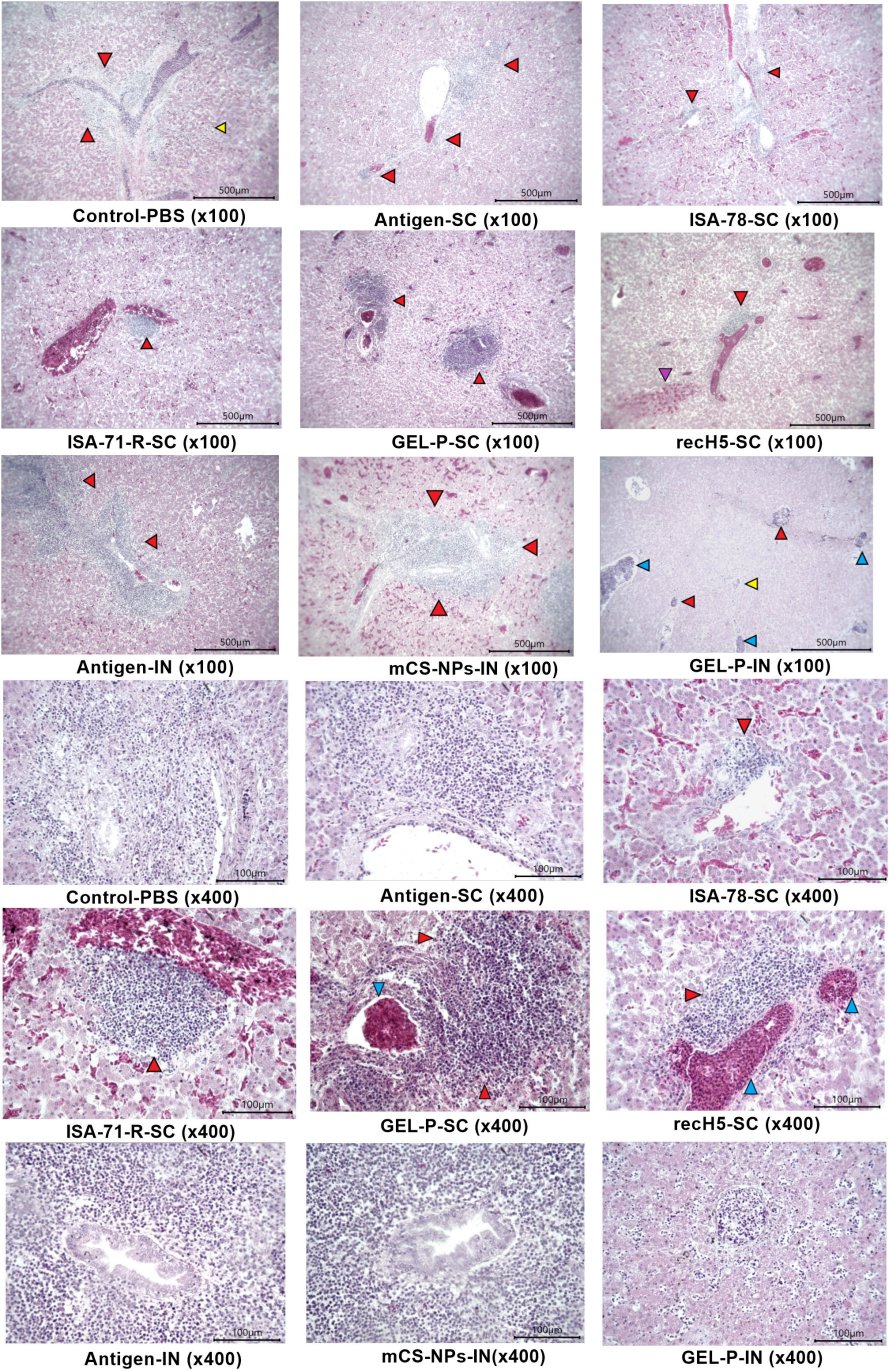
Histological analysis of liver tissue. Control-PBS (×100): severe foci of periportal necrosis (red arrows) and parenchyma hepatocyte necrosis (yellow arrow); antigen-SC (×100): severe periportal necrosis; ISA-78-SC (×100): diffuse hemorrhage and foci of necrosis with lymphocytic infiltration near the portal tract (red arrows); ISA-71-R-SC (×100): focal moderate foci of periportal necrosis (red arrow) and diffuse hemorrhages in the parenchyma; GEL-P-SC (×100): foci of centrilobular and periportal necrosis (red arrows); recH5-SC (×100): focal hemorrhage in the parenchyma (purple arrow) and periportal necrosis (red arrow) of hepatocytes; antigen-IN (×100): foci of periportal necrosis; mCS-NPs-IN (×100): strongly marked focus of periportal necrosis (red arrow) and diffuse hemorrhages in the parenchyma; GEL-P-IN (×100): hepatocyte necrosis in the parenchyma (yellow arrow), periportal and centrilobular foci of necrosis (red arrows), and vessels filled with lymphocytes and macrophages (blue arrow); control-PBS (×400): severe foci of periportal hepatocyte necrosis; antigen-SC (×400): severe foci of necrosis at the bile duct; ISA-78-SC (×400): mild centrilobular necrosis of hepatocytes with lymphoid cells (red arrow). GEL-P-SC (×400): foci of marked centrilobular necrosis (red arrows) and erythrostasis (blue arrow) in the vessel. GEL-P-IN (×400): hepatocyte necrosis and lymphocytes in sinusoidal spaces. ISA-71-R-SC (×400): focal moderate foci of periportal necrosis (red arrow) and diffuse hemorrhages in the parenchyma; recH5-SC (×400): moderate periportal necrosis of hepatocytes (red arrow) and erythrostasis in the vessel (blue arrow). Antigen-IN (×400): a pronounced focus of periportal necrosis; mCS-NPs-IN (×400): strongly marked focus of necrosis at the bile duct; hematoxylin–eosin staining.

Control infected birds showed notable pathological changes near the parabronchi, including necrosis of the atrial epithelium, transudate accumulation in the parabronchial cavity, erythrostasis within vessels, and low-grade lymphocytic infiltration (**Figure 8**), indicating localized tissue damage and inflammatory responses associated with infection. The histopathological analysis of liver specimens of control infected birds also revealed significant changes, including enlargement of sinusoidal spaces, focal necrosis in the parenchyma, particularly in the periportal region and near the centrilobular vein, as well as areas of hemorrhage and erythrostasis within vessels (**Figure 9**).

**Figure 10:**
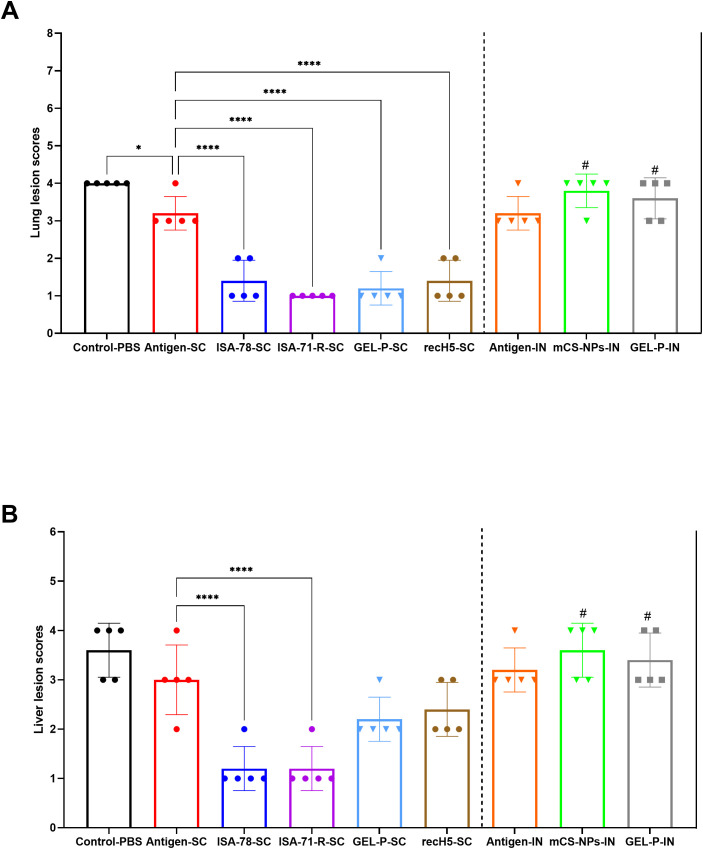
Histopathological scoring of lung and liver lesions in H5N1-infected chickens. **(A)** Lung lesion scores. **(B)** Liver lesion scores. The histological scale used was as follows: 0 (no changes; 0%), 1 (mild inflammation; <25%), 2 (moderate inflammation; 26%–50%), 3 (pronounced inflammation; 51%–75%), and 4 (severe inflammation; 76%–100%). Data are mean ± SD titer of five chickens in each group. Statistical differences between groups were assessed using Holm–Šídák’s multiple comparisons test. For all comparisons, *P* < 0.05 was considered a significant difference. *P = 0.0233; ****P < 0.0001. A second intranasal immunization was administered to groups GEL-P-IN and mCS-NPs-IN at 14 days after prime vaccination.

SC vaccines, particularly ISA-78-SC, GEL-P-SC, ISA-71-R-SC, and the commercial vaccine rec-H5-SC, reduced the pathology caused by H5N1 infection (**Figure 10A**) as evidenced by significantly lower lesion scores (*P* < 0.0001) compared to the group that received antigen alone SC, which displayed the highest lesion scores after the SC and IN PBS control groups. In the IN groups, including GEL-P-IN and mCS-NPs-IN, the lesion scores were not significantly different from those of the group that received antigen alone via the IN route.

For liver lesions, only the SC groups ISA-78-SC and ISA-71-R-SC showed effective protection (*P* < 0.0001) (**Figure 10B**). IN administration of GEL-P-IN or mCS-NPs-IN provided no protection against liver lesions.

This confirmed that the SC route of administration was superior to IN for preventing lung and liver damage following H5N1 infection, with ISA-78-SC and ISA-71-R-SC formulations showing the greatest protection.

## 4 Discussion

H5Nx HPAI is a devastating infection of poultry in Asian, European, North African, and Central and Northern American countries. An increasing number of HPAI outbreaks and the establishment of endemicity in many countries have resulted in an increased use of vaccination as a tool in control programs (30). According to the World Organisation for Animal Health (WOAH), more than 30 countries have resorted to vaccination against HPAI since 2005, including Mexico, China, Guatemala, Honduras, El Salvador, Egypt, and European Union (31). Among the various techniques for vaccine strain development, reverse genetics is the most extensively utilized for creating non-pathogenic DIVA (differentiation of infected from vaccinated animals) marker vaccines (32, 33).

The present study was aimed at evaluating different adjuvants (oil-based, aqueous, and nanoparticles) and delivery routes (parenteral and mucosal) for a reverse-genetics-based vaccine based on IDCDC-RG71A (H5N8; clade 2.3.4.4b), developed as part of the WHO’s Pandemic Influenza Preparedness Framework (19). The selection of the IDCDC-RG71A (H5N8) strain for vaccine development is supported by its high genetic homology in the HA gene with the circulating H5N1 field virus in KZ (clade 2.3.4.4b), sharing 97% identity, as reported in our previous studies (2). This high level of HA gene similarity ensures that the HA of the vaccine strain closely resembles the circulating virus, thereby conferring the high chance of protection against local H5N1 strains. Additionally, the inclusion of a neuraminidase (NA) gene from a different subtype than the target H5N1 field virus confers DIVA compatibility to the H5N8 vaccine. This design allows for the serological differentiation of H5N8-vaccinated birds from those naturally infected with H5N1 by using N1-based serological assays (30), which is in line with the heterologous NA DIVA strategy (32).

These study results confirm the successful inactivation, safety, and tolerability of the formaldehyde-inactivated H5N8 vaccine in SAN chickens. The absence of adverse effects alongside consistent growth metrics underscores the tolerability and safety of the tested adjuvant formulations, which were comparable to the commercial recH5-SC (VOLVAC^®^ B.E.S.T AI+ND) vaccine (33). Notably, the experimental adjuvanted vaccine formulations induced much higher HI activity than the commercial recH5-SC vaccine which induced very little HI activity, highlighting the importance of the adjuvants that we used to maximize vaccine immunogenicity. This result is consistent with the findings reported by Kandeil et al. (2018) showing low HI titers induced by commercial HPAI vaccines (34).

The study demonstrated that the single-dose SC H5N8 formulations, particularly those containing Montanide mineral oil adjuvants (ISA-78, ISA-71-R) or aqueous adjuvant (GEL-P-SC), provided the most robust protection against H5N1 infection and clinical disease, alongside the commercial oil-based recH5-SC vaccine, while also significantly reducing virus shedding via the respiratory and digestive tracts by day 2 post-challenge compared to the antigen-SC group (antigen administered subcutaneously without adjuvant). These findings align with data from Kuruppuarachchi et al. (2022) (35), which showed that an oil-adjuvanted inactivated H5N6 vaccine completely protected the chickens from the lethal infection with homologous H5N6 and heterologous H5N1 HPAI viruses. In their study, no viral shedding was observed from either the trachea or cloaca on day 3 post-challenge with the H5N6 vaccine, whereas our H5N8 vaccine achieved similar results by day 2 post-challenge. The histological analysis further confirmed the effectiveness of SC-adjuvanted vaccine formulations, especially those with ISA-78 and ISA-71-R, in protecting chickens from lung and liver lesions caused by H5N1 infection, whereas all of the IN H5N8 vaccine formulations (mCS-NPs-IN and GEL-P-IN) provided minimal protection even after two doses.

The ISA-71-R SC formulation achieved the highest serum anti-influenza IgY levels, whereas only the IN groups, particularly GEL-P-IN, induced anti-influenza IgA production in the lung and trachea. While a positive correlation was observed between serum influenza-binding IgY, HI activity, and protection, the mucosal IgA responses showed no correlation with protection. It is generally suggested that IgA antibody subtype is the primary isotype induced at the mucosal surfaces and could be involved in protecting animals from infections by influenza viruses (36). In studies by Hwang et al. (2011) (37), it was shown that in chickens immunized with a single dose of an oil-adjuvanted inactivated H5N1 vaccine, IgG was predominantly induced over IgA in the sera, with the authors suggesting that IgG plays the most important role in protecting the immunized chickens against the lethal H5N1 infections.

There were no significant differences in CD8+ T cell responses between any groups. CD4+ T cell responses showed high variability and were only significantly increased in group ISA-78-SC compared to the SC antigen alone group, showing a correlation with protection in this group only. These results partially confirm other reports (38) showing that the major correlate of H5N1 protection in animals is systemic HI activity and Th1-cytokine-secreting CD4+ T cells.

These findings underscore the importance of adjuvants and SC delivery to maximize HPAI vaccine efficacy.

The limitations of this study include several factors that may affect the generalizability and robustness of the findings. One primary limitation is the challenge model itself, as only a single H5N1 strain was used for the post-vaccination challenge, which may not fully represent the broad diversity of H5 avian influenza strains. This approach may limit the generalizability of the findings to other clades. The DIVA capability of this vaccine may be limited in regions with extensive H5Nx circulation, as cross-reactive antibodies could reduce assay specificity. Further validation is needed to ensure effectiveness in diverse settings. Furthermore,antigen-specific cytokine production, such as IFN-gamma, was not assessed in this study. The absence of an IFN-gamma ELISPOT assay limits the understanding of the functional T-cell responses elicited by the vaccines. Future studies will incorporate this assay to provide a more comprehensive evaluation of T-cell activation and its role in protective immunity. An additional limitation is the lackof IgG assessment, which could further clarify the protective mechanisms of our vaccine. While IgY is the primary immunoglobulin in birds, future studies will evaluate IgG responses to enhance our understanding of immunity against HPAI virus. Another limitation involves the reliance on IN delivery in certain groups, which showed lower efficacy compared to SC routes. This outcome suggests a need for optimization in adjuvant selection and administration routes for mucosal vaccines to enhance immune responses. Additionally, the study was conducted with SAN chickens, which may exhibit different immune responses than poultry populations with prior exposures to low pathogenic avian influenza viruses. This factor should be considered when interpreting the real-world applicability of the vaccine efficacy results. The study also employed relatively small group sizes, potentially impacting the statistical power and robustness of the findings. Furthermore, the study was not repeated, necessitating independent replication to confirm the efficacy and consistency of the results. Another limitation is thelack of assessment for the durability of protection, as the study focused on short-term outcomes post-vaccination. This leaves open questions about the long-term immunity provided by the vaccine formulations. Although safety and immunogenicity were rigorously assessed over a 35-day period, longer-term studies are warranted to evaluate sustained immunity and potential impacts on poultry production metrics. An additional limitation is the lack of mucosal immune response evaluation in IN vaccination groups, which restricts understanding of localized immunity. This experiment will be repeated in future studies with nasal secretion sampling to assess mucosal antibody responses. Finally, apart from HI activity, the study did not measure functional antibodies, such as neutralizing antibodies (MN) or neuraminidase inhibition (NI) activity. These assays are crucial to assess the quality of immune responses and understand the protective mechanisms of the vaccines. Future studies will incorporate MN and NI assays to comprehensively evaluate the functional antibody responses elicited by the vaccine formulations. These limitations together highlight the need for future studies with larger sample sizes, repeat trials, and extended observation periods to assess the durability of protection,which would provide a more comprehensive understanding of vaccine efficacy.

In conclusion, this study demonstrates the promise of H5N8 strain IDCDC-RG71A, developed using reverse genetics, to protect against H5N1 infection in poultry. When combined with ISA-78 or ISA-71-R mineral oil adjuvants, these SC vaccines demonstrated robust efficacy with high survival rates and control of clinical signs (abnormally high temperature), reduced the virus shedding, and prevented the lung and liver lesions caused by HPAI H5N1. These results underscore the importance of adjuvants and SC delivery to maximize avian influenza vaccine efficacy and offer valuable insights for the development of potent, DIVA-compatible vaccines that could significantly enhance biosecurity and disease management in regions affected by endemic HPAI.

## Data Availability

The raw data supporting the conclusions of this article will be made available by the authors, without undue reservation.
