# Supplementary material for: Evaluation of safety, immunogenicity, and efficacy of inactivated reverse-genetics-based H5N8 highly pathogenic avian influenza virus vaccine with various adjuvants via parenteral and mucosal routes in chickens
Source: Front Immunol. 2025 Mar 20;16:1539492. doi: 10.3389/fimmu.2025.1539492 (PMC11965622; doi:10.3389/fimmu.2025.1539492)
Supplement: Supplementary file 1 [file Table1.docx]

Supplementary Material

**Supplementary Table 1.** Mean body weight in pullets in the safety test

| Group | Number of chickens | Days after vaccination | | | | | |
| --- | --- | --- | --- | --- | --- | --- | --- |
|  |  | 0 | 7 | 14 | 21 | 28 | 35 |
| ISA-78-SC | 10 | 242 ± 21 | 284 ± 25 | 360 ± 21 | 409 ± 23 | 475 ± 19 | 552 ± 21 |
| ISA-71-R-SC | 10 | 225 ± 17 | 292 ± 20 | 342 ± 23 | 421 ± 18 | 482 ± 15 | 564 ± 18 |
| GEL-P-SC | 10 | 242 ± 15 | 277 ± 14 | 354 ± 29 | 408 ± 22 | 486 ± 25 | 560 ± 26 |
| recH5-SC | 10 | 252 ± 17 | 285 ± 16 | 332 ± 26 | 410 ± 17 | 491 ± 21 | 565 ± 17 |
| Antigen-SC | 10 | 239 ± 17 | 283 ± 20 | 345 ± 20 | 413 ± 12 | 486 ± 14 | 552 ± 14 |
| mCS-NPs-IN | 10 | 216 ± 19 | 287 ± 17 | 341 ± 18 | 408 ± 25 | 485 ± 15 | 556 ± 24 |
| GEL-P-IN | 10 | 239 ± 20 | 286 ± 15 | 346 ± 19 | 405 ± 19 | 480 ± 18 | 564 ± 22 |
| Antigen-IN | 10 | 240 ± 20 | 284 ± 23 | 332 ± 25 | 400 ± 24 | 488 ± 28 | 559 ± 24 |
| Control-PBS | 10 | 249 ± 22 | 293 ± 20 | 357 ± 26 | 426 ± 22 | 510 ± 26 | 590 ± 17 |

**
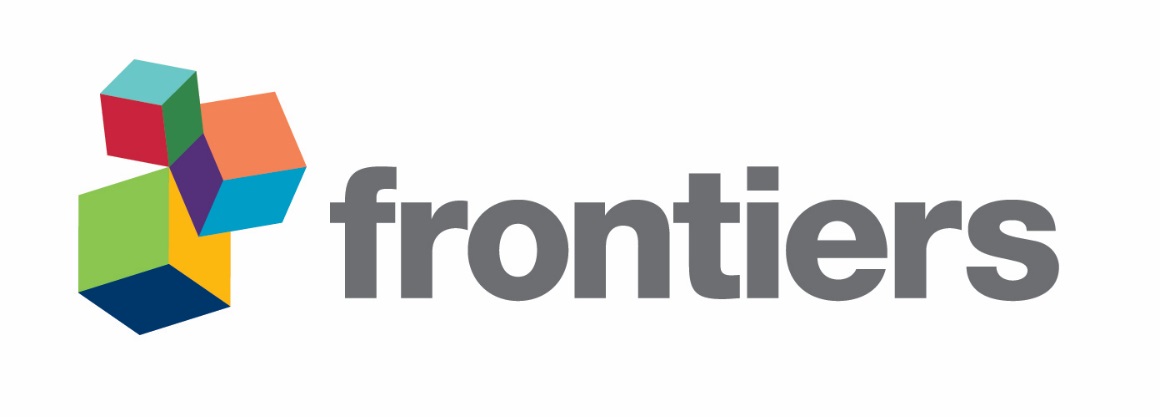
**
