# Supplementary material for: Evaluation of safety, immunogenicity, and efficacy of inactivated reverse-genetics-based H5N8 highly pathogenic avian influenza virus vaccine with various adjuvants via parenteral and mucosal routes in chickens
Source: Front Immunol. 2025 Mar 20;16:1539492. doi: 10.3389/fimmu.2025.1539492 (PMC11965622; doi:10.3389/fimmu.2025.1539492)
Supplement: Supplementary file 2 [file Table2.docx]

Supplementary Material

**Supplementary Table 2.** Day of death and percentage protection for each group of the vaccinated and control chickens

| Group | Days post-challenge | | | | | | | | | | Number of deaths | Protection (%) |
| --- | --- | --- | --- | --- | --- | --- | --- | --- | --- | --- | --- | --- |
|  | 1 | 2 | 3 | 4 | 5 | 6 | 7 | 8 | 9 | 10 |  |  |
| ISA-78-SC |  |  |  |  |  |  |  |  |  |  | 0 | 100% |
| ISA-71-R-SC |  |  |  |  |  |  |  |  |  |  | 0 | 100% |
| GEL-P-SC |  |  |  |  |  |  |  |  |  |  | 0 | 100% |
| recH5-SC |  |  |  |  |  |  |  |  |  |  | 0 | 100% |
| Antigen-SC |  |  | 3 | 1 |  |  |  |  |  |  | 4 | 20% |
| mCS-NPs-IN |  | 1 | 2 |  |  |  |  |  |  |  | 3 | 40% |
| GEL-P-IN |  | 1 | 3 |  | 1 |  |  |  |  |  | 5 | 0% |
| Antigen-IN |  | 1 | 4 |  |  |  |  |  |  |  | 5 | 0% |
| Control-PBS |  | 2 | 3 |  |  |  |  |  |  |  | 5 | 0% |

**
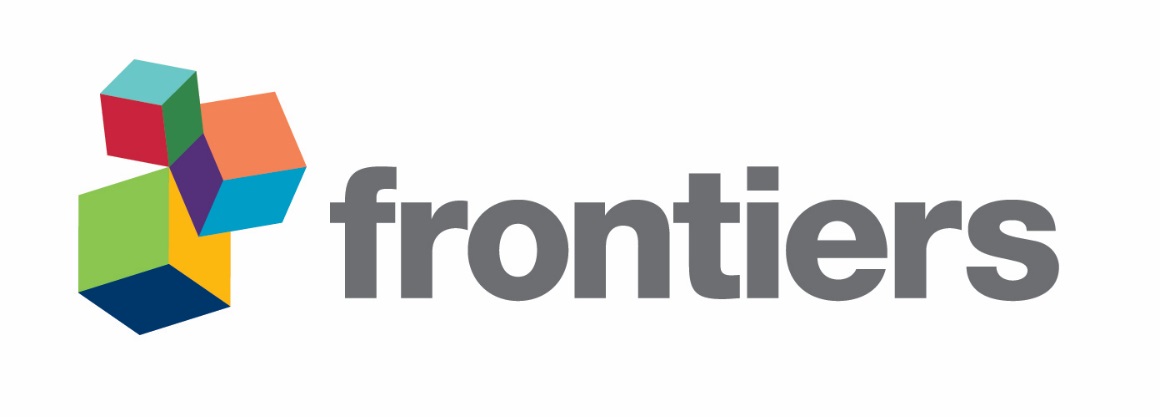
**
