# Supplementary material for: Evaluation of safety, immunogenicity, and efficacy of inactivated reverse-genetics-based H5N8 highly pathogenic avian influenza virus vaccine with various adjuvants via parenteral and mucosal routes in chickens
Source: Front Immunol. 2025 Mar 20;16:1539492. doi: 10.3389/fimmu.2025.1539492 (PMC11965622; doi:10.3389/fimmu.2025.1539492)
Supplement: Supplementary file 3 [file Table3.docx]

Supplementary Material

**Supplementary Table 3.** Occurrence of abnormally high temperature by day post-challenge and overall incidence within each vaccine group

| Group | Days post-challenge | | | | | | | | | | Number of chickens  with ≥42.3°С | Percentage | |
| --- | --- | --- | --- | --- | --- | --- | --- | --- | --- | --- | --- | --- | --- |
|  | 1 | 2 | 3 | 4 | 5 | 6 | 7 | 8 | 9 | 10 |  |  |  |
| ISA-78-SC |  |  |  |  |  |  |  |  |  |  | 0 | 0% |  |
| ISA-71-R-SC |  |  |  |  |  |  |  |  |  |  | 0 | 0% |  |
| GEL-P-SC |  | 1 | 1 |  |  |  |  |  |  |  | 1 | 20% |  |
| recH5-SC |  |  |  |  |  |  |  |  |  |  | 0 | 0% |  |
| Antigen-SC |  | 2 | 1 |  |  |  |  |  |  |  | 3 | 60% |  |
| mCS-NPs-IN | 1 | 2 |  |  |  |  |  |  |  |  | 3 | 60% |  |
| GEL-P-IN | 1 | 3 |  | 1 |  |  |  |  |  |  | 5 | 100% |  |
| Antigen-IN | 1 | 2 |  |  |  |  |  |  |  |  | 3 | 60% |  |
| Control-PBS | 3 | 3 |  |  |  |  |  |  |  |  | 5 | 100% |  |

**
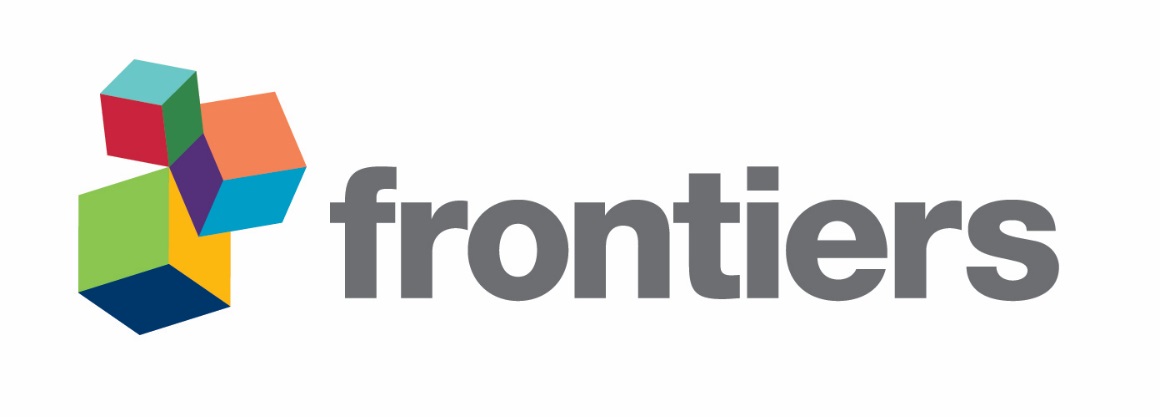
**
